# Azukisapogenol Triterpene Glycosides from *Oxytropis chiliophylla* Royle

**DOI:** 10.3390/molecules23102448

**Published:** 2018-09-25

**Authors:** Jun Wang, Hongshuai Yang, Yang Liu, Kelsang Norbo, Kewu Zeng, Mingbo Zhao, Hong Liang, Pengfei Tu, Qingying Zhang

**Affiliations:** State Key Laboratory of Natural and Biomimetic Drugs and Department of Natural Medicines, School of Pharmaceutical Sciences, Peking University Health Science Center, 38 Xueyuan Road, Beijing 100191, China; 18134280270@163.com (J.W.); hongshuai@bjmu.edu.cn (H.Y.); 1311210122@bjmu.edu.cn (Y.L.); kelsang815@163.com (K.N.); ZKW@bjmu.edu.cn (K.Z.); zmb@bjmu.edu.cn (M.Z.); lianghong@bjmu.edu.cn (H.L.); pengfeitu@bjmu.edu.cn (P.T.)

**Keywords:** *Oxytropis*, *Oxytropis chiliophylla*, azukisapogenol triterpene glycosides, anti-inflammatory activity

## Abstract

Eight azukisapogenol triterpene glycosides, including five new compounds, oxychiliotriterpenosides A–E (**1**–**5**), two new methyl glucuronide derivatives that proved to be artifacts, oxychiliotriterpenoside E-glucuronic acid methyl ester (**6**) and myrioside B-glucuronic acid methyl ester (**7**), and a known one, myrioside B (**8**), was isolated from the aerial part of *Oxytropis chiliophylla* Royle. Their structures were elucidated based on extensive spectroscopic analyses and chemical methods. Triterpene glycosides were first obtained from *O. chiliophylla*, and those containing a galactose unit (**1**, **2**, **5** and **6**) and diglucosidic or triglucosidic linkage at C-29 (**1**–**4**), were reported from *Oxytropis* species for the first time, which might be recognized as a chemotaxonomic feature of *O. chiliophylla*. All isolated compounds were evaluated for their anti-inflammatory activities against NO production using lipopolysaccharide (LPS)-induced RAW 264.7 cells, but no compounds showed potent inhibition on NO production.

## 1. Introduction

**Figure 1:**
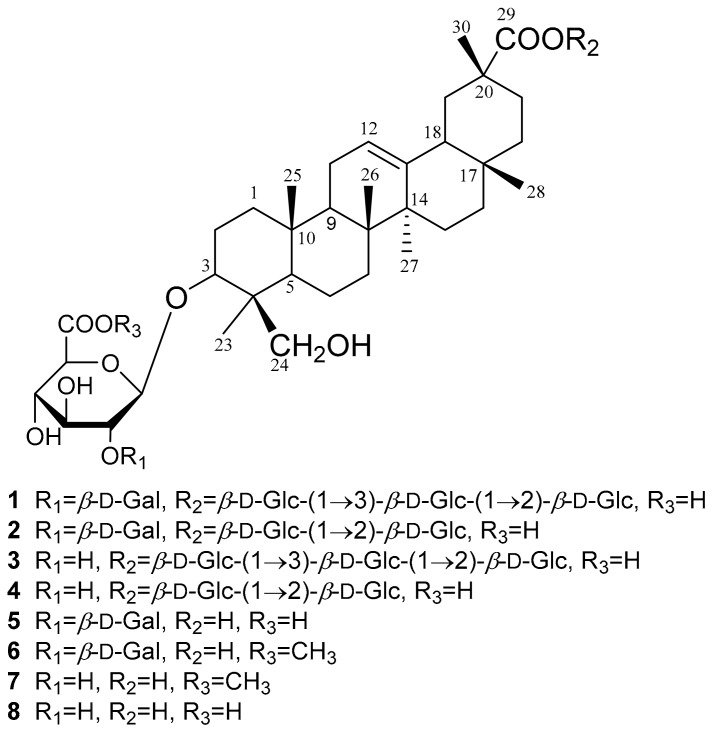
Chemical structures of compounds **1**–**8.**

*Oxytropis chiliophylla* Royle (Leguminosae) is officially documented as one of the botanical origin of Tibetan medicine “Er-Da-Xia” that is known as “King of Herbs”, and is widely used for the treatment of inflammation, fever, and bleeding [1,2]. Inhabited at 2800–5200 m altitude, *O. chiliophylla* is mainly distributed in ravines, on slopes, in steppe meadows, and shrubberies in Tibet and Xinjiang Uygur Autonomous Regions in China [3]. However, chemical and pharmacological investigations of *O. chiliophylla* were rare. Yao et al. reported the isolation of three flavonoids, one triterpenoid, and four other compounds from the herb [4], and our previous phytochemical investigation on the aerial part of *O. chiliophylla* led to the isolation of fourteen rhamnocitrin glycosides [5] and ten isomeric cyclobutane and cyclohexene-containing chalcone dimers [6]. As part of our ongoing research for novel and bioactive constituents from *O. chiliophylla*, we report herein the isolation and structural elucidation of eight azukisapogenol triterpene glycosides, including five new compounds, oxychiliotriterpenosides A–E (**1**–**5**), two new methyl ester derivatives that proved to be artifacts, oxychiliotriterpenoside E-glucuronic acid methyl ester (**6**) and myrioside B-glucuronic acid methyl ester (**7**), and a known one, myrioside B (**8**) (Figure 1). Triterpene glycosides were isolated from *O. chiliophylla* for the first time, and those containing a galactose unit (**1**, **2**, **5** and **6**) and diglucosidic or triglucosidic linkage at C-29 (**1**–**4**), which might be chemotaxonomic markers of *O. chiliophylla*, were reported from *Oxytropis* species for the first time. Furthermore, their anti-inflammatory activities against NO production using LPS-induced RAW 264.7 cells were also evaluated.

## 2. Results and Discussion

The dried aerial parts of *O. chiliophylla* were pulverized and were, in turn, extracted with 95% EtOH and 50% EtOH. The combined and concentrated 95% EtOH and 50% EtOH extracts were suspended in water and then partitioned successively with petroleum ether, EtOAc and *n*-BuOH. The *n*-BuOH extract was separated by repeated chromatography on macroporous resin, silica gel, and octadecyl silane (ODS), and semi-preparative RP-HPLC to afford eight azukisapogenol glycosides, including five new compounds (**1**–**5**), two new methyl ester derivatives that proved to be artifacts (**6**–**7**), and one known compound (**8**). The structures of new compounds (**1**–**7**) were identified by comprehensive spectroscopic analyses and chemical methods. The known compound (**8**) was identified as myrioside B, which was previously reported from *O. myriophylla* [7], by comparison of the spectral data.

**Figure 2:**
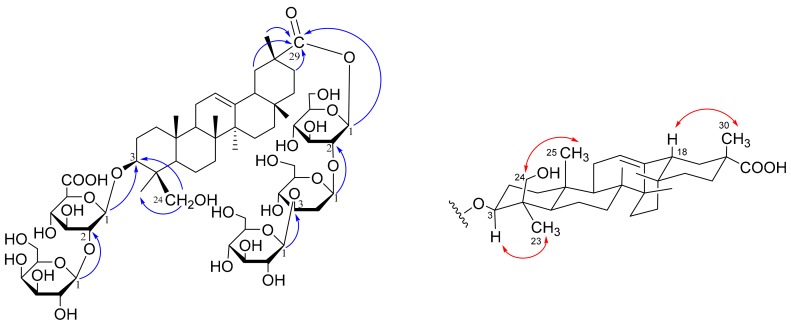
Key HMBC and NOE correlations of compound **1**.

Compound **1** was obtained as a white and amorphous powder. Its molecular formula was determined to be C_60_H_96_O_30_ by the cationated molecular ion peak [M + Na]^+^ at *m/z* 1319.5876 (calcd for C_60_H_96_O_30_Na, 1319.5884) in positive high resolution electrospray ionization mass spectrometry (HRESIMS) (Figure S1). The ^1^H and ^13^C-NMR data (Table 1 and Table 2) indicated **1** to be an olean-12-ene triterpene glycoside containing five sugar units, evidenced by the characteristic signals of six angular methyl signals at *δ*_H_ 0.67, 0.84, 0.87, 1.08, 1.32, and 1.49 (each 3H, s), an olefinic proton at *δ*_H_ 5.21 (1H, overlapped), five anomeric protons at *δ*_H_ 6.24 (1H, d, *J* = 8.0 Hz), 5.49 (1H, d, *J* = 7.5Hz), 5.46 (1H, d, *J* = 7.7 Hz), 5.20 (1H, d, *J* = 7.8 Hz), and 4.98 (1H, d, *J* = 7.5 Hz), and the corresponding olefinic carbons at *δ*_C_ 144.44, 123.38 and anomeric carbons at *δ*_C_ 105.87, 105.55, 104.97, 104.92, and 94.12 according to the HSQC spectrum (Figure S5). In addition, resonances at *δ*_C_ 177.98 and 173.17 implied the presence of two carboxyl acid or ester groups. Detailed comparison of the ^1^H and ^13^C NMR data with those reported in the literature showed close similarity between the aglycones of **1** and azukisapogenol 3-*O*-[α-l-arabinopyranosyl-(1→2)-β-d-glucuronopyranosyl]-29-*O*-glucopyranoside [8], which is indicative of the same aglycone of the two compounds and was further confirmed by 2D NMR correlations, including correlation spectroscopy (^1^H-^1^H COSY), heteronuclear single quantum coherence (HSQC), heteronuclear multiple-bond correlation (HMBC), heteronuclear single quantum coherence-total correlation spectroscopy (HSQC-TOCSY) and nuclear overhauser effect spectroscopy (NOESY) (Figure 2 and Figures S4–S9). Thus, the aglycone of **1** was assigned as azukisapogenol, i.e., 3*β*, 24-dihydroxy-12-en-olean-29-oic acid.

The sugar moieties were initially proposed as one glucuronic acid (GlcA) unit and four glucose (Glc) residues, which are common for *Oxytropis* triterpene glycosides. However, detailed examination of the selected 1D TOCSY (Figure S9) and HSQC-TOCSY (Figure S7) allowed the total assignment of all sugar signals and showed that the multiplicities of H-3 [*δ*_H_ 4.07 (dd, *J* = 9.8, 2.8 Hz)] and H-4 [*δ*_H_ 4.40 (br d, 2.8)] of a sugar unit were markedly different from the triplets (*J* = ~9.0 Hz) of Glc H-3 and Glc H-4 due to axial-axial coupling. The double doublet and broad doublet of H-3 and H-4 of the sugar unit indicated that its 4-OH should be axial-oriented, and thus the sugar should be galactose (Gal). HPLC analysis of the arylthiocarbamate derivatives of sugar units [9], revealing three peaks corresponding to D-Gal, D-Glc, and D-GlcA, respectively, further confirmed the above deduction. The GlcA residue was linked to C-3 of the aglycone according to the HMBC correlation of *δ*_H_ 4.98 (GlcA H-1) with *δ*_C_ 91.00 (C-3), while the Gal was placed at GlcA C-2 by the key HMBC correlations of *δ*_H_ 5.49 (Gal H-1) with *δ*_C_ 81.14 (GlcA C-2). Additionally, the connection among the remaining three glucose units and aglycone was determined by the heteronuclear multiple-bond correlation (HMBC) correlations between *δ*_H_ 6.24 (Glc_1_ H-1) and *δ*_C_ 177.98 (Aglycone C-29), between *δ*_H_ 5.46 (Glc_2_ H-1) and *δ*_C_ 80.60 (Glc_1_ C-2), and between *δ*_H_ 5.20 (Glc_3_ H-1) and *δ*_C_ 88.24 (Glc_2_ C-3) (Figure 2). The *β*-anomeric configuration of each sugar was assigned from the large coupling constant of each anomeric proton. Thus, the structure of compound **1** was identified as azukisapogenol 3-*O*-[β-d-galactopyranosyl-(l→2)-β-d-glucuronopyranosyl]-29-*O*-β-d-glucopyranosyl-(l→3)-β-d-glucopyranosyl-(l→2)-β-d-glucopyranoside. Compound **1** is a new compound and was named oxychiliotriterpenoside A.

Compound **2**, isolated as a white and amorphous powder, had the molecular formula of C_54_H_86_O_25_ as deduced by the positive HRESIMS ([M + Na]^+^ *m/z* 1157.5388, calcd for C_54_H_86_O_25_Na, 1157.5356) (Figure S10). Its ^1^H and ^13^C NMR data (Table 1 and Table 2) resembled to those of **1** except for the lack of a set of signals for one glucose unit, which was in good accordance with the significant upfield shift of Glc_2_ C-3 compared to **1** (*δ*_C_ 78.15 in **2**; *δ*_C_ 88.24 in **1**) and the molecular formula of **2** showing C_6_H_10_O_5_ less than that of **1**. Acid hydrolysis and coupling patterns of anomeric protons confirmed the presence of *β*-d-galactose, β-d-glucose, and β-d-glucuronic acid. Thus, the structure of **2** was identified as azukisapogenol 3-*O*-[β-d-galactopyranosyl-(l→2)-β-d-glucuronopyranosyl]-29-*O*-β-d-glucopyranosyl-(l→2)-β-d-glucopyranoside, which was unambiguously confirmed by 2D NMR data (Figures S13–S16). Compound **2** is a new compound and was named oxychiliotriterpenoside B.

Compound **3**, a white and amorphous powder, had a molecular formula of C_54_H_86_O_25_, the same as **2**, by positive HRESIMS at *m/z* 1157.5375 [M + Na]^+^ (calcd for C_54_H_86_O_25_Na, 1157.5356) (Figure S17). Differed from **2**, the distinction between **3** and **1** was found by the absence of a set of signals corresponding to one *β*-d-galactose, and this was consistent with the acid hydrolysis results that yielded only d-glucose and d-glucuronic acid and the significant upfield shift of GlcA C-2 compared to **1** (*δ*_C_ 75.69 in **3**; *δ*_C_ 81.14 in **1**). Coupling pattern of anomeric protons confirmed the *β*-anomeric configuration of each sugar residue. The sequence of the sugar chains and their connections with the aglycone as determined by HMBC correlations (Figure S22) were the same as **1**. Consequently, the structure of **3** was defined as azukisapogenol 3-*O*-[β-d-glucuronopyranosyl]-29-*O*-β-d-glucopyranosyl-(l→3)-β-d-glucopyranosyl-(l→2)-β-d-glucopyranoside and named oxychiliotriterpenoside C.

Compound **4** was isolated as a white amorphous powder. Its molecular formula of C_48_H_76_O_20_, C_6_H_10_O_5_ less than that of **3**, was determined by the cationated molecular ion peak [M+Na]^+^ at *m/z* 995.4836 (calcd for C_48_H_76_O_20_Na, 995.4828) in positive HRESIMS (Figure S24). The ^1^H and ^13^C NMR data (Table 1 and Table 2) exhibited characteristic signals of azukisapogenol glycoside with three sugar units consisting of one GlcA and two Glc moieties. Further acid hydrolysis and 2D NMR experiments (Figures S27–S30) confirmed the structure of **4** to be azukisapogenol 3-*O*-[β-d-glucuronopyranosyl]-29-*O*-β-d-glucopyranosyl-(l→2)-β-d-glucopyranoside. Compound **4** is a new compound and was named oxychiliotriterpenoside D.

Compound **5** had the molecular formula of C_42_H_66_O_15_, according to [M + Na]^+^ at *m/z* 833.4296 (calcd for C_42_H_66_O_15_Na, 833.4299) in positive HRESIMS (Figure S31). The ^1^H and ^13^C NMR data of **5** (Table 1 and Table 2), exhibiting characteristic signals of azukisapogenol glycoside with two sugar units, differed from those of compounds **1**–**4** not only in the lack of signals for sugar residues, but also in the significant downfield shift of C-29 (*δ*_C_ 181.45 in **5**; *δ*_C_ 177.98, 178.00, 178.08, and 178.11 in **1**–**4**), implying the absence of glycosidic esterification at C-29 in **5**. Acid hydrolysis and coupling patterns of the anomeric protons confirmed the presence of β-d-galactose and β-d-glucuronic acid. The key HMBC correlations of *δ*_H_ 4.88 (GlcA H-1) with *δ*_C_ 90.81 (Aglycone C-3), and *δ*_H_ 5.43 (Gal H-1) with *δ*_C_ 81.16 (GlcA C-2) assigned the structure of **5** to be azukisapogenol 3-*O*-β-d-galactopyranosyl-(l→2)-β-d-glucuronopyranoside, which was confirmed by 2D NMR (Figures S34–S37). Compound **5** is a new compound and was named oxychiliotriterpenoside E.

Compound **6**, a white amorphous powder, had a molecular formula of C_43_H_68_O_15_ by HRESIMS analysis ([M + Na]^+^ *m/z* 847.4457, calcd for C_43_H_68_O_15_Na, 847.4456) (Figure S38). The ^1^H and ^13^C NMR data (Table 1 and Table 2) highly resembled those of **5**, except for the occurrence of one more methoxyl group (*δ*_H_ 3.74, *δ*_C_ 52.46) in **6**. Moreover, the molecular formula of **6**, showing one more CH_2_ than that of **5**, and the upfield shift of GlcA C-6 (*δ*_C_ 170.55 in **6**; *δ*_C_ 172.63 in **5**) supported **6** to be a methyl glucuronide derivative of **5**. The HMBC correlation of *δ*_H_ 3.74 (OCH_3_) with *δ*_C_ 170.55 (GlcA C-6) (Figure S43) further confirmed the linkage of OCH_3_ to GlcA C-6. Consequently, compound **6** was characterized as azukisapogenol 3-*O*-β-d-galactopyranosyl-(l→2)-β-d-6-*O*-methyl-glucuronopyranoside, i.e., oxychiliotriterpenoside E glucuronic acid methyl ester.

Compound **7**, a white amorphous powder, gave the molecular formula of C_37_H_58_O_10_ as deduced by HRESIMS analysis ([M + Na]^+^ *m/z* 685.3925, calcd for C_37_H_58_O_10_Na, 685.3928) (Figure S44). The ^1^H and ^13^C NMR data (Table 1 and Table 2) indicated **7** to be an azukisapogenol glucuronide, in which the glucuronic acid was methyl esterified evidenced by the characteristic signals of OCH_3_ (*δ*_H_ 3.74, *δ*_C_ 52.43) and CO (*δ*_C_ 171.03). Detailed analysis of the 1D and 2D NMR data (Table 1 and Table 2 and Figures S45–S49) confirmed the structure of **7** to be azukisapogenol 3-*O*-*β*-d-6-*O*-methyl glucuronopyranoside, i.e., myrioside B glucuronic acid methyl ester.

**Figure 3:**
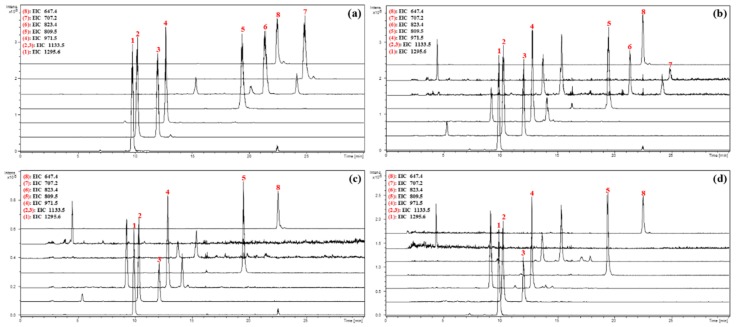
Extract ion chromatogram (EICs) of the reference compounds **1**–**8** and different extracts of *O. chiliophylla* by extracting *m/z* 1295.6, 1133.5, 971.5, 809.5, 823.5, 707.5 and 647.5. (**a**) Reference compounds; (**b**) MeOH extract; (**c**) EtOH extract; and (**d**) Water extract.

Compounds **6** and **7**, the methyl glucuronide derivatives of **5** and myrioside B, seemed to be artifacts formed during the isolation procedure. Thus, 1.0 g dried powder of *O. chiliophylla* was extracted under reflux by 10 mL methanol, 10 mL ethanol and 10 mL water, respectively. Then the methanol, ethanol and water extracts, as well as the reference substances of **1**–**8**, were analyzed using LC-MS by extracting the corresponding deprotonated molecular ion peak [M − H]^−^ of **1**–**6** and **8** and cationated [M + HCOO]^−^ of **7** with the mass accuracy <20 ppm. In the EIC chromatogram, compounds **1**–**8** exhibited peaks with retention time of 9.7, 10.2, 12.0, 12.7, 19.4, 21.4, 24.8, and 22.4 min, respectively. As we predicted, **6** and **7** were obviously detected in the methanol extract but not detected in the ethanol and water extracts, while **1**–**5** and **8** can be obviously detected in all the extracts (Figure 3). Consequently, it can be concluded that compounds **1**–**5** and **8** are naturally existing in *O. chiliophylla*, while **6** and **7** are artifacts that might be derived from **5** and myrioside B, respectively.

Triterpene glycosides, an important group of secondary metabolites with a variety of biological activities, such as anti-inflammatory, antiviral, cytotoxic, and anti-fugal [10,11,12,13], are well-known to be one of the major bioactive constituents of *Oxytropis* species. Seventeen olean-12-ene and one cycloartane triterpene glycosides, with soyasapogenol B, soyasapogenol E, and azukisapogenol as the common aglycones, have been reported from *O. myriophylla* [7], *O. glabra* [14], and *O. kansuensis* [15]. The sugar portion consists of one to four sugar units including GlcA, Glc, rhamnose (Rha), arabinose (Ara), and xylose (Xyl), where the GlcA unit, substituted with other sugars at its C-2, C-4, or C-6, is directly linked to aglycone via C-3 position, whereas the bidesmosidic glycosides identified from *Oxytropis* usually have a monoglucosidic linkage at C-29 of the azukisapogenol aglycone. Triterpene glycosides have not been reported from *O. chiliophylla* to date, and the current study is the first to report the separation of triterpene glycosides from the herb. All the obtained triterpene glycosides from *O. chiliophylla* here are derived from the same aglycone of azukisapogenol, which is common in *Oxytropis* genus [7,14]. Azukisapogenol and its glycosylated derivatives were firstly isolated from *Vigna angulariz* [16,17,18] and subsequently found in *O. glabra* [14], *O. myriophylla* [7], and *Trifolium hybridum* [8], all of which belonged to the same subfamily of Papilionoideae (Leguminosae). Concerning the sugar moieties of the obtained triterpene glycosides from *O. chiliophylla*, GlcA, Glc, and Gal are identified as the sugar units, with the glycosidic chains of GlcA at C-3 and Glc at C-29 respectively that is common for the triterpene glycosides previously reported from *Oxytropis* speciecs. However, Gal, usually linked to C-2 of GlcA, was identified as new sugar units (**1**, **2**, **5**, and **6**) of azukisapogenol glycosides, and the bidesmosidic glycosides, having a diglucosidic or triglucosidic linkage at C-29 (**1**–**4**), are reported from *Oxytropis* for the first time. Thus azukisapogenol glycosides, especially those with a galactose unit at GlcA C-2, might be recognized as a chemotaxonomic feature of *O. chiliophylla*. Further investigation of the triterpene glycosides of *Oxytropis* species is necessary for a better understanding of their chemical and biological roles in these plants.

All the isolated compounds were further evaluated for their anti-inflammatory activity through LPS-induced NO production in RAW 264.7 cells. Unfortunately, no compounds showed potent inhibition on NO production, which might be due to the glycosylation that decreases the lipophilic nature and makes it difficult to penetrate cell membrane.

## 3. Experimental Section

### 3.1. General Experimental Procedures

Optical rotations were measured with Rudolph Research Analytical Autopol IV automatic polarmeter (Rudolph Research Analytical, Wilmington, MA, USA). NMR spectra were recorded on a Bruker AVANCE III-400 or 600 (Bruker Corporation, Billerica, MA, USA) with TMS as internal standard. HRESIMS was carried out on a Waters Xevo G2 Q-TOF spectrometer fitted with an ESI source (Waters Corporation, Milford, MA, USA). HPLC analysis was performed on an Agilent 1260 LC system (Agilent Technologies, USA) with a Phenomenex column (250 × 4.6 mm, 5 μm). Preparative HPLC separations were performed on an Alltech semi-preparative HPLC instrument (Alltech Corporation, Chicago, IL, USA) equipped with a Grace Alltime C18 column (250 × 22 mm, 5 μm). Column chromatography (CC) was performed on Diaion HP20 (200–300 mesh, Mitsubishi Chemical Co., Tokyo, Japan), silica gel (200–300 mesh; Qingdao Marine Chemical, Inc., Qingdao, China), ODS-A (50 μm, YMC Co. Ltd., Kyoto, Japan), and Sephadex LH-20 (GE Healthcare Bio-Science AB, Uppsala, Sweden). Analytical TLC was carried out on silica GF_254_ (10–40 μm; QingDao Marine Chemical, Inc., Qingdao, China), and spots were observed by a UV light and 10% H_2_SO_4_-EtOH reagent. HPLC grade solvents, used for HPLC analysis and preparation, were purchased from Fisher Scientific International (Fair Lawn, NJ, USA), and deionized water was purified by Milli-Q Synthesis A10 (Bedford, MA, USA). Other solvents, used for extraction and isolation, were of analytical grade and purchased from Beijing Tongguang Chemicals (Beijing, China). All chemicals were purchased from J & K Co. Ltd. (Beijing, China).

### 3.2. Plant Material

The whole plant of *O. chiliophylla* was collected from Nagarze County, Tibet Autonomous Region, China, in September 2012, and was authenticated by Associate Professor Ying-Tao Zhang, Department of Natural Medicines, School of Pharmaceutical Science, Peking University Health Science Center. A voucher specimen (No 20120901) was deposited in the Herbarium of Department of Natural Medicines, School of Pharmaceutical Sciences, Peking University Health Science Center.

### 3.3. Extraction and Isolation

The dried aerial part of *O. chiliophylla* (10.0 kg) was pulverized and in turn extracted with 95% EtOH and 50% EtOH for three times. After removal of solvent under reduced pressure, the residue was suspended in water and then partitioned successively with petroleum ether, EtOAc and *n*-BuOH, respectively. The *n*-BuOH extract (250 g) was chromatographed on Diaion HP20 macroporous absorbent resin using an EtOH/H_2_O gradient (0%, 20%, 40%, 60%, 80% and 95% EtOH) to obtain six fractions (DK1~DK6). DK4 (eluted with 60% EtOH, 80 g) was subjected to silica gel CC eluted with CH_2_Cl_2_/CH_3_OH gradient system (10:1, 5:1, 3:1, 1:1, and 1:5) to give five fractions (DK4-1~DK4-5). DK4-3 (13.2 g) was chromatographed on ODS CC with a gradient system of MeOH/H_2_O (30, 50, 70, and 100% MeOH) to yield four sub-fractions (DK4-3-A~DK4-3-D). Further purification of DK4-3-D (2.0 g) by CC on silica gel with EtOAc/MeOH/H_2_O gradient system (45:2:1, 30:2:1, and 15:2:1) afforded compounds **5** (200 mg), **6** (100 mg), **7** (20 mg), and **8** (10 mg). DK4-5 (20.0 g) was chromatographed on silica gel CC eluted with EtOAc/MeOH/H_2_O gradient system (30:2:1, 15:2:1, 8:2:1) to give four sub-fractions (DK4-5-A~DK4-5-D). Further separation of DK4-5-D by isocratic preparative HPLC (MeCN-0.08% formic acid water, 33:67) gave compounds **1** (1.0 g), **2** (500 mg), **3** (200 mg), and **4** (20 mg).

*Oxychiliotriterpenoside A(***1***)*: white amorphous powder; ${\left[\alpha \right]}_{D}^{25}$ +20.0 (c 0.1, MeOH); UV (MeOH) *λ*_max_ 210 nm; IR (KBr) *ν*_max_ 3419, 2920, 1748, 1730, 1647, 1455, 1436, 1382, 1026, and 576 cm^−1^; HRESIMS (positive mode) *m/z* 1319.5876 [M + Na]^+^ (calcd for C_60_H_96_O_30_Na, 1319.5884); ^1^H NMR and ^13^C NMR (Pyr-*d*_5_) data, see Table 1 and Table 2.

*Oxychiliotriterpenoside B (***2***)*: white amorphous powder; ${\left[\alpha \right]}_{D}^{25}$ +30.0 (c 0.1, MeOH); UV (MeOH) *λ*_max_ 210 nm; IR (KBr) *ν*_max_ 3428, 2919, 1748, 1730, 1648, 1606, 1454, 1381, 1029, and 576 cm^−1^; HRESIMS (positive mode) *m/z* 1157.5388 [M + Na]^+^ (calcd for C_54_H_86_O_25_Na, 1157.5356); ^1^H NMR and ^13^C NMR (Pyr-*d*_5_) data, see Table 1 and Table 2.

*Oxychiliotriterpenoside C (***3***)*: white amorphous powder; ${\left[\alpha \right]}_{D}^{25}$ +10.0 (c 0.1, MeOH); UV (MeOH) *λ*_max_ 210 nm; IR (KBr) *ν*_max_ 3410, 2921, 1741, 1610, 1463, 1381, 1030, and 581 cm^−1^; HRESIMS (positive mode) *m/z* 1157.5375 [M + Na]^+^ (calcd for C_54_H_86_O_25_Na, 1157.5356); ^1^H NMR and ^13^C NMR (Pyr-*d*_5_) data, see Table 1 and Table 2.

*Oxychiliotriterpenoside D (***4***)*: white amorphous powder; ${\left[\alpha \right]}_{D}^{25}$ +30.0 (c 0.1, MeOH); UV (MeOH) *λ*_max_ 210 nm; IR (KBr) *ν*_max_ 3356, 2927, 1744, 1609, 1464, 1381, 1079, 1028, 659, and 581 cm^−1^; HRESIMS (positive mode) *m/z* 995.4836 [M + Na]^+^ (calcd for C_48_H_76_O_20_Na, 995.4828); ^1^H NMR and ^13^C NMR (Pyr-*d*_5_) data, see Table 1 and Table 2.

*Oxychiliotriterpenoside E (***5***)*: white amorphous powder; ${\left[\alpha \right]}_{D}^{25}$ +30.0 (c 0.1, MeOH); UV (MeOH) *λ*_max_ 210 nm; IR (KBr) *ν*_max_ 3415, 2924, 1714, 1467, 1382, 1257, 1042, and 576 cm^−1^; HRESIMS (positive mode) *m/z* 833.4296 [M + Na]^+^ (calcd for C_42_H_66_O_15_Na, 833.4299); ^1^H NMR and ^13^C NMR (Pyr-*d*_5_) data, see Table 1 and Table 2.

*Oxychiliotriterpenoside E glucuronic acid methyl ester (***6***)*: white amorphous powder; ${\left[\alpha \right]}_{D}^{25}$ +10.0 (c 0.1, MeOH); UV (MeOH) *λ*_max_ 210 nm; IR (KBr) *ν*_max_ 3419, 2920, 1747, 1605, 1455, 1379, 1025, and 523 cm^−1^; HRESIMS (positive mode) *m/z* 847.4457 [M + Na]^+^ (calcd for C_43_H_68_O_15_Na, 847.4456); ^1^H NMR and ^13^C NMR (Pyr-*d*_5_) data, see Table 1 and Table 2.

*Myrioside B glucuronic acid methyl ester (***7***)*: amorphous, white powder; ${\left[\alpha \right]}_{D}^{25}$ +10.0 (c 0.1, MeOH); UV (MeOH) *λ*_max_ 210 nm; IR (KBr) *ν*_max_ 3421, 2924, 1746, 1456, 1380, 1028, and 578 cm^−1^; HRESIMS (positive mode) *m/z* 685.3925 [M + Na]^+^ (calcd for C_37_H_58_O_10_Na,685.3928); ^1^H NMR and ^13^C NMR (Pyr-*d*_5_) data, see Table 1 and Table 2.

### 3.4. Determination of Absolute Configurations of Sugar Moieties

Compounds **1**−**7** (each 2.0 mg) were hydrolyzed with 3 M HCl for 2 h at 120 °C. The reaction product was dissolved in H_2_O after evaporation, and then extracted with CH_2_Cl_2_ for three times. After being concentrated to dryness, the aqueous residue was added into 0.5 mL anhydrous pyridine containing 2.0 mg L-cysteine methyl ester hydrochloride, and heated at 60 °C for 1 h. Then, *O*-tolyl (5 μL) was added and heated at 60 °C for another 1 h. Subsequently, the reaction mixture was directly analyzed by HPLC under the following conditions: An Agilent 1260 chromatograph equipped with a Phenomenex column (250 × 4.6 mm, 5 μm); column temperature: 35 °C; mobile phase: isocratic elution of 23% MeCN-H_2_O (*v*:*v*) containing 0.08% formic acid; flow rate: 0.8 mL/min; UV detection wavelength: 250 nm. The *t*_R_ values (min) of standard monosaccharide derivatives prepared in the same way were 18.9 (D-Gal), 21.7 (D-Glc), and 22.5 (D-GlcA), respectively. By comparison of the retention times, D-Gal, D-Glc, and D-GlcA were identified from compounds **1**, **2**, **5**, and **6**, while D-Glc and D-GlcA were identified from compounds **3**, **4**, and **7**.

### 3.5. LC-MS Analysis

#### 3.5.1. Chromatographic and MS Conditions

An Agilent 1200 series HPLC system and an Agilent 6320 ion trap MS equipped with an ESI source were used for sample analysis. Separations were performed by a Phenomenex column (250 × 4.6 mm, 5 μm) at 35 °C with a mobile phase consisting of 0.05% formic acid in water (A) and acetonitrile (B). The gradient elution conditions of mobile phase were as follows: 0–10 min, 30–40% B; 10–30 min, and 40–100% B. The flow rate was 1.0 mL/min, and the injection volume was 10 μL. The HPLC effluent was introduced into the ESI source in a post-column splitting ratio of 1:5. The spectra were recorded in the range of *m/z* = 100–1400. The mass setting parameters were as follows: Fragmentation amplitude, 0.5 V, nebulizer 45.0 psi, and the gas temperature was 350 °C with gas flow of 10.0 L/min. 

#### 3.5.2. Preparation of Sample Solutions

Approximately 1.0 g of powdered aerial part of *O. chiliophylla* was extracted under reflux for 6 h by 10 mL MeOH, 10 mL EtOH and 10 mL water, respectively, and filtered through a 0.22 μm filter membrane prior to injection into the LC-MS system.

#### 3.5.3. Preparation of Reference Solutions

Compounds **1**–**8** were weighed accurately and dissolved in methanol to obtain solutions of 100 μg/mL as the reference solutions.

#### 3.5.4. LC-MS Identification of Compounds **1**–**8**

The methanol, ethanol and water extracts of *O. chiliophylla*, as well as the reference substances **1**–**8,** were analyzed by LC-MS. Ionization was achieved using ESI in the negative mode. The data were collected and then processed by MSD Trap Control Version 6.1 workstation.

#### 3.5.5. Cell Culture

Mouse RAW 264.7 monocytic cells were from the Cell Bank of Peking Union Medical College (Beijing, China) grown in DMEM medium (Hyclone, Waltham, MA, USA), and supplemented with 10% fetal bovine serum (Hyclone), penicillin (100 U/mL), and streptomycin (100 μg/mL) in a humidified incubator containing 95% air and 5% CO_2_ at 37 °C.

### 3.6. Anti-Inflammatory Activity Assay

The anti-inflammatory effects were investigated by detecting the productions of nitric oxide (NO), a major regulatory molecule involved in inflammatory response, in cell culture supernatants. Briefly, RAW 264.7 cells (5.0 × 10^4^ cells per well) were treated with lipopolysaccharide (LPS from *Escherichia coli*, serotype 055:B5, 1 μg/mL) and different compounds for 24 h. Then, cell culture supernatants (100 μL) were collected and reacted with 100 μL of Griess reagent (0.1% naphthylethylene diamine dihydrochloride/1% sulfanilamide/2% phosphoric acid). After incubation for 10 min at room temperature, the optical density was detected at 540 nm using a microplate reader. Sodium nitrite was used as a standard curve in the assay.

## Figures and Tables

**Table 1:** ^1^H NMR Data for Compounds **1**–**7** in Pyr-*d*_5._

Position	1	2	3	4	5	6	7
Aglycone 1	1.34–1.39 ^a^ 0.81–0.87 ^a^	1.26–1.32 ^a^ 0.75–0.83 ^a^	1.47–1.51 ^a^ 0.93–0.98 ^a^	1.40–1.45 ^a^ 0.88–0.94 ^a^	1.26–1.35 ^a^ 0.69–0.77 ^a^	1.30–1.38 ^a^ 0.72–0.77 ^a^	1.43–1.49 ^a^ 0.83–0.89 ^a^
2	2.25–2.32 ^a^ 1.81–1.90 ^a^	2.14–2.23 ^a^ 1.76–1.86 ^a^	2.29 m 1.95–1.99 ^a^	2.15–2.20 ^a^ 1.94–1.99 ^a^	2.15–2.21 ^a^ 1.79–1.87 ^a^	2.09–2.14 ^a^ 1.68–1.74 ^a^	2.12–2.19 ^a^ 1.69–1.75 ^a^
3	3.43 dd (10.5, 3.5)	3.29–3.37 ^a^	3.57–3.62 ^a^	3.53 m	3.30–3.39 ^a^	3.30–3.38 ^a^	3.57 dd (10.9, 4.3)
5	0.73–0.78 ^a^	0.72 br d (10.7)	0.86 br d (9.4)	0.81–0.85 ^a^	0.76 br d (10.8) ^a^	0.77 br d (9.2)	0.87–0.92 ^a^
6	1.47–1.52 ^a^ 1.16–1.30 ^a^	1.45–1.52 ^a^ 1.19–1.25 ^a^	1.62 m 1.35–1.39 ^a^	1.60–1.64 ^a^ 1.35–1.39 m	1.46–1.52 ^a^ 1.20–1.29 ^a^	1.47–1.54 ^a^ 1.19–1.27 ^a^	1.65–1.70 ^a^ 1.41–1.47 ^a^
7	1.26–1.32 ^a^ 1.13–1.25 ^a^	1.26–1.32 ^a^ 1.13–1.19 ^a^	1.35–1.39 ^a^ 1.21 m	1.36–1.40 ^a^ 1.22 m	1.28–1.35 ^a^ 1.16–1.24 ^a^	1.29–1.36 ^a^ 1.17–1.24 ^a^	1.42–1.47 ^a^ 1.25–1.31 ^a^
9	1.43–1.49 ^a^	1.42–1.48 ^a^	1.52–1.56 ^a^	1.52–1.58 ^a^	1.45–1.51 ^a^	1.46–1.54 ^a^	1.55–1.60 ^a^
11	1.81–1.87 ^a^ 1.65–1.72 ^a^	1.77–1.90 ^a^ 1.62–1.74 ^a^	1.93 m 1.73 m	1.91–1.98 ^a^ 1.71–1.77 ^a^	1.70–1.78 (2H)	1.70–1.78 (2H)	1.83 m (2H)
12	5.21 ^a^	5.22 br s	5.27 br s	5.27 br s	5.22 br s	5.22 br s	5.27 br s
15	1.61–1.69 ^a^ 0.83–0.88 ^a^	1.60–1.69 ^a^ 0.82–0.90 ^a^	1.66–1.70 ^a^ 0.88–0.92 ^a^	1.65–1.72 ^a^ 0.89–0.93 ^a^	1.65–1.72 ^a^ 0.85–0.92 ^a^	1.65–1.69 ^a^ 0.82–0.86 ^a^	1.71–1.77 ^a^ 0.80–0.90 ^a^
16	1.97–2.01 ^a^ 0.75–0.81 ^a^	2.02 br t (12.2) ^a^ 0.75–0.81 ^a^	2.01–2.06 ^a^ 0.78–0.82 ^a^	2.06 m 0.79–0.83 ^a^	2.09–2.14 ^a^ 0.82–0.86 ^a^	2.09–2.14 ^a^ 0.82–0.86 ^a^	2.12–2.19 ^a^ 0.83–0.89 ^a^
18	2.06 dd (14.1, 3.5)	2.06 dd (13.8, 3.2)	2.11 dd (13.8, 3.5)	2.06–2.13 ^a^	2.07–2.14 ^a^	2.07–2.14 ^a^	2.14–2.20 ^a^
19	2.37 t (13.6) 1.60 br d (12.8)	2.38 t (13.3) 1.59 br d (11.4)	2.38 t (13.6) 1.65–1.70 ^a^	2.42 t (13.6) 1.63–1.69 ^a^	2.49 t (13.2) 1.63–1.70 ^a^	2.49 t (13.2) 1.63–1.70 ^a^	2.58 t (13.2) 1.69–1.75 ^a^
21	2.12 br td (12.4, 2.6) 1.63–1.70 ^a^	2.13 br t (12.4) ^a^ 1.63–1.69 ^a^	2.18 br t (12.4) 1.63–1.68 ^a^	2.15–2.21 ^a^ 1.66–1.71 ^a^	2.16–2.24 ^a^ 1.62–1.71 ^a^	2.16–2.24 ^a^ 1.67–1.73 ^a^	2.29 br t (12.5) 1.70–1.76 ^a^
22	1.45–1.54 ^a^ 1.26–1.33 ^a^	1.44–1.51 ^a^ 1.26–1.32 ^a^	1.47–1.51 ^a^ 1.32 m	1.49–1.54 ^a^ 1.32 m	1.47–1.53 ^a^ 1.31–1.37 ^a^	1.46–1.54 ^a^ 1.31–1.37 ^a^	1.53–1.60 1.35–1.42
23	1.32 s	1.29 s	1.52 s	1.51 s	1.28 s	1.28 s	1.50 s
24	4.21–4.28 3.35 d (11.2)	4.20–4.27 ^a^ 3.29–3.36 ^a^	4.34–4.38 ^a^ 3.64 d (11.2)	3.64 d (11.0) 4.33–4.38 ^a^	4.20–4.27 ^a^ 3.27–3.38 ^a^	4.20–4.26^a^ 3.30–3.38 ^a^	4.39 d (11.0) 3.68 d (11.0) ^a^
25	0.67 s	0.65 s	0.78 s	0.77 s	0.69 s	0.70 s	0.83 s
26	0.84 s	0.84 s	0.90 s	0.90 s	0.87 s	0.88 s	0.95 s
27	1.08 s	1.09 s	1.13 s	1.15 s	1.18 s	1.18 s	1.26 s
28	0.87 s	0.86 s	0.89 s	0.89 s	0.91 s	0.91 s	0.95 s
30	1.49 s	1.45 s	1.50 s	1.52 s	1.43 s	1.43 s	1.56 s
Sugars (C-3)							
Glu A 1	4.98 d (7.5)	4.89 d (5.5)	5.13 d (7.0)	5.09 d (5.2)	4.88 d (6.2)	4.86 d (6.2)	5.14 d (7.4)
2	4.31 dd (8.7, 7.5)	4.26–4.31 ^a^	4.04–4.08 ^a^	4.09 m	4.25–4.33 ^a^	4.24–4.28 ^a^	4.10 dd (8.7, 7.4)
3	4.35 dd (8.9, 8.7)	4.31–4.36 ^a^	4.27–4.31 ^a^	4.29–4.36 m	4.28–4.33 ^a^	4.25–4.30 ^a^	4.30 dd (9.0, 8.7)
4	4.51 dd (9.6, 8.9)	4.45–4.52 ^a^	4.50–4.54 ^a^	4.53–4.58 ^a^	4.41–4.49 ^a^	4.30–4.37 ^a^	4.51 dd (9.7, 9.0)
5	4.63 d (9.6)	4.51–4.56 ^a^	4.69 d (8.2)	4.68 br s	4.50–4.55 ^a^	4.43–4.48	4.68 d (9.7)
6-OCH_3_						3.74 s	3.74 s
Gal 1	5.49 d (7.5)	5.48 d (7.0)			5.43 d (7.0)	5.44 d (7.1)	
2	4.47 t (9.7, 7.5)	4.45 ^a^			4.40–4.46 ^a^	4.40–4.44	
3	4.07 dd (9.7, 2.8)	4.05 br d (8.2)			4.02 br d (8.8)	4.04 br d (9.2)	
4	4.40 br d (2.8)	4.36–4.42 ^a^			4.33–4.39 ^a^	4.35–4.40 ^a^	
5	3.94–3.98 ^a^	3.92–3.97 ^a^			3.92 m	3.93 m	
6	4.43–4.47 ^a^ 4.30–4.35 ^a^	4.39–4.46 ^a^ 4.28–4.34 ^a^			4.37–4.45 ^a^ 4.25–4.30 ^a^	4.30–4.50 ^a^ 4.22–4.30 ^a^	
Sugars (C-29)							
Glc_1_ 1	6.24 d (8.0)	6.23 d (8.0)	6.27 d (8.0)	6.28 d (8.0)			
2	4.40 t (8.7)	4.37–4.43 ^a^	4.44 t (8.7)	4.42–4.49 ^a^			
3	4.27 t (9.1)	4.26–4.31 ^a^	4.27–4.31 ^a^	4.32–4.33 ^a^			
4	4.19 t (9.1)	4.17–4.25 ^a^	4.22 ^a^	4.22–4.28 ^a^			
5	3.91 m	3.90–3.96 ^a^	3.91–3.95 ^a^	3.94–4.00 ^a^			
6	4.35–4.39 ^a^ 4.26–4.30 ^a^	4.33–4.38 ^a^ 4.24–4.29 ^a^	4.38–4.42 ^a^ 4.28–4.33 ^a^	4.38–4.44 ^a^ 4.30–4.35 ^a^			
Glc_2_ 1	5.46 d (7.7)	5.42 d (7.6)	5.51 d (7.7)	5.49 d (7.6)			
2	3.99 t (8.4)	3.97 ^a^	4.00 ^a^	4.02 t (8.4)			
3	4.16 t (9.0)	4.17–4.23 ^a^	4.14–4.18 ^a^	4.21–4.27 ^a^			
4	4.07 t (9.0)	4.16–4.22 ^a^	4.04–4.08 ^a^	4.21–4.26 ^a^			
5	3.89 m	3.92–3.97 ^a^	3.89–3.93 ^a^	3.95–4.01 ^a^			
6	4.47 br d (11.2) 4.33 dd (11.2, 4.4)	4.49–4.56 ^a^ 4.37–4.42 ^a^	4.32–4.36 ^a^ 4.49–4.53 ^a^	4.43–4.48 ^a^ 4.55–4.59 ^a^			
Glc_3_ 1	5.20 d (7.8)		5.22 d (7.5)				
2	4.02 t (8.4)		4.03–4.07 ^a^				
3	4.20 t (9.0)		4.21–4.25 ^a^				
4	4.12 t (9.0)		4.14–4.18 ^a^				
5	3.98 m		3.97–4.02 ^a^				
6	4.53 br d (11.6) 4.25 dd (11.6, 5.8)		4.50–4.54 ^a^ 4.25–4.30 ^a^				

^a^ Overlapped with other signals.

**Table 2:** ^13^C NMR Data for Compounds **1**–**7** in Pyr-*d_5_*.

Position	1	2	3	4	5	6	7
Aglycone							
1	38.73	38.61	38.85	38.75	38.77	38.78	38.92
2	26.80	26.80	27.00	27.06	26.87	26.86	27.21
3	91.00	90.86	89.33	89.16	90.81	91.97	89.39
4	44.01	43.98	44.64	44.64	44.02	44.05	44.78
5	56.28	56.19	56.32	56.26	56.25	56.31	56.37
6	18.85	18.80	19.07	19.04	18.88	18.88	19.18
7	33.13	33.12	33.32	33.34	33.21	33.24	33.47
8	40.17	40.13	40.30	40.28	40.21	40.21	40.40
9	47.75	47.69	47.78	47.75	47.86	47.90	48.00
10	36.66	36.61	36.83	36.81	36.67	36.68	36.92
11	24.16	24.14	24.25	24.27	24.25	24.27	24.43
12	123.38	123.40	123.60	123.62	123.12	122.95	123.33
13	144.44	144.35	144.48	144.40	144.84	144.96	144.94
14	41.90	41.90	42.02	42.04	42.05	42.06	42.22
15	26.58	26.55	26.65	26.62	26.65	26.68	26.76
16	27.32	27.29	27.40	27.39	27.41	27.45	27.55
17	32.83	32.81	32.92	32.92	32.98	33.01	33.14
18	46.38	46.35	46.48	46.50	46.67	46.80	46.86
19	40.91	40.90	41.10	41.02	41.69	41.82	41.84
20	43.51	43.50	43.62	43.61	43.05	43.22	43.22
21	29.63	29.64	29.61	29.70	30.11	30.22	30.26
22	36.33	36.28	36.41	36.37	36.72	36.87	36.84
23	23.01	22.89	23.58	23.55	22.88	22.87	23.63
24	63.80	63.71	63.55	63.51	63.71	63.68	63.56
25	15.87	15.85	15.73	15.71	15.91	15.90	15.78
26	16.99	16.98	17.11	17.13	17.05	17.06	17.22
27	26.26	26.22	26.35	26.29	26.31	26.31	26.40
28	28.48	28.45	28.57	28.55	28.62	28.66	28.75
29	177.98	178.00	178.08	178.11	181.45	181.53	181.62
30	19.44	19.39	19.39	19.44	20.20	20.29	20.32
Sugar (C-3)							
Glu A 1	104.97	105.03	106.48	106.65	105.06	105.10	106.85
2	81.14	80.92	75.69	75.66	81.16	80.75	75.66
3	78.38	78.36	78.33	78.41	78.31	78.06	78.22
4	73.21	73.18	73.85	73.81	73.17	72.86	73.60
5	77.78	77.71	77.91	78.07	77.78	77.12	77.73
6	173.17	172.98	174.12	173.42	172.63	170.55	171.03
6-OCH_3_						52.46	52.43
Gal 1	105.55	105.41			105.57	105.38	
2	73.81	73.76			73.79	73.72	
3	75.63	75.57			75.55	75.54	
4	71.24	71.14			71.21	71.17	
5	77.42	77.33			77.35	77.30	
6	62.83	62.73			62.87	62.75	
Sugar (C-29)							
Glc_1_ 1	94.12	94.26	94.20	94.38			
2	80.60	80.95	80.33	80.94			
3	78.50	78.36	78.60	78.51			
4	70.84	70.83	70.96	70.96			
5	79.58	79.52	79.70	79.67			
6	62.14	62.10	62.22	62.21			
Glc_2_ 1	104.92	105.68	104.82	105.76			
2	75.24	76.56	75.25	76.68			
3	88.24	78.15	88.33	78.28			
4	70.04	71.90	70.21	72.03			
5	78.50	78.76	78.56	78.89			
6	62.88	63.08	63.01	63.23			
Glc_3_ 1	105.87		105.94				
2	75.71		75.77				
3	78.42		78.46				
4	71.88		71.93				
5	78.81		78.86				
6	62.80		62.82				

## Supplementary Materials

The HRESIMS, ID and 2D NMR spectra of compounds **1**–**7** are available online.
