# Supplementary material for: Azukisapogenol Triterpene Glycosides from Oxytropis chiliophylla Royle
Source: Molecules. 2018 Sep 25;23(10):2448. doi: 10.3390/molecules23102448 (PMC6222378; doi:10.3390/molecules23102448)
Supplement: Supplementary file 1 [file molecules-23-02448-s001.pdf]

Supporting information

Azukisapogenol Triterpene Glycosides from *Oxytropis chiliophylla* Royle

Jun Wang†, Hongshuai Yang†, Yang Liu, Norbo Kelsang, Kewu Zeng, Zhao Mingbo, Hong Liang, Pengfei Tu, Qingying Zhang *

State Key Laboratory of Natural and Biomimetic Drugs and Department of Natural Medicines, School of Pharmaceutical Sciences, Peking University Health Science Center, 38 Xueyuan Road, Beijing 100191, P. R. China

†The authors contribute equally.

*Correspondence: qyzhang@hsc.pku.edu.cn; Tel/Fax: +86-10-8280-1725

Contents

Figure S1. HRESIMS spectrum of oxychiliotriterpenoside A (**1**)

Figure S2. ^{1}H NMR spectrum of oxychiliotriterpenoside A (**1**) in Pyr-d_5

Figure S3. ^{13}C NMR spectrum of oxychiliotriterpenoside A (**1**) in Pyr-d_5

Figure S4. ^{1}H-^{1}H COSY spectrum of oxychiliotriterpenoside A (**1**) in Pyr-d_5

Figure S5. HSQC spectrum of oxychiliotriterpenoside A (**1**) in Pyr-d_5

Figure S6. HMBC spectrum of oxychiliotriterpenoside A (**1**) in Pyr-d_5

Figure S7. HSQC-TOCSY spectrum of oxychiliotriterpenoside A (**1**) in Pyr-d_5

Figure S8. NOESY spectrum of oxychiliotriterpenoside A (**1**) in Pyr-d_5

Figure S9. Selected 1D TOCSY spectrum of oxychiliotriterpenoside A (**1**) in Pyr-d_5

Figure S10. HRESIMS spectrum of oxychiliotriterpenoside B (**2**)

Figure S11. ^{1}H NMR spectrum of oxychiliotriterpenoside B (**2**) in Pyr-d_5

Figure S12. ^{13}C NMR spectrum of oxychiliotriterpenoside B (**2**) in Pyr-d_5

Figure S13. ^{1}H-^{1}H COSY spectrum of oxychiliotriterpenoside B (**2**) in Pyr-d_5

Figure S14. HSQC spectrum of oxychiliotriterpenoside B (**2**) in Pyr-d_5

Figure S15. HMBC spectrum of oxychiliotriterpenoside B (**2**) in Pyr-d_5

Figure S16. HSQC-TOCSY spectrum of oxychiliotriterpenoside B (**2**) in Pyr-d_5

Figure S17. HRESIMS spectrum of oxychiliotriterpenoside C (**3**)

Figure S18. ^{1}H NMR spectrum of oxychiliotriterpenoside C (**3**) in Pyr-d_5

Figure S19. ^{13}C NMR spectrum of oxychiliotriterpenoside C (**3**) in Pyr-d_5

Figure S20. ^{1}H-^{1}H COSY spectrum of oxychiliotriterpenoside C (**3**) in Pyr-d_5

Figure S21. HSQC spectrum of oxychiliotriterpenoside C (**3**) in Pyr-d_5

Figure S22. HMBC spectrum of oxychiliotriterpenosideC (**3**) in Pyr-d_5

Figure S23. HSQC-TOCSY spectrum of oxychiliotriterpenoside C (**3**) in Pyr-d_5

Figure S24. HRESIMS spectrum of oxychiliotriterpenoside D (**4**)

Figure S25. ^{1}H NMR spectrum of oxychiliotriterpenoside D (**4**) in Pyr-d_5

Figure S26. ^{13}C NMR spectrum of oxychiliotriterpenoside D (**4**) in Pyr-d_5

Figure S27. ^{1}H-^{1}H COSY spectrum of oxychiliotriterpenoside D (**4**) in Pyr-d_5

Figure S28. HSQC spectrum of oxychiliotriterpenoside D (**4**) in Pyr-d_5

Figure S29. HMBC spectrum of oxychiliotriterpenosideD (**4**) in Pyr-d_5

Figure S30. HSQC-TOCSY spectrum of oxychiliotriterpenoside D (**4**) in Pyr-d_5

Figure S31. HRESIMS spectrum of oxychiliotriterpenoside E (**5**)

Figure S32. ^{1}H NMR spectrum of oxychiliotriterpenoside E (**5**) in Pyr-d_5

Figure S33. ^{13}C NMR spectrum of oxychiliotriterpenoside E (**5**) in Pyr-d_5

Figure S34. ^{1}H-^{1}H COSY spectrum of oxychiliotriterpenoside E (**5**) in Pyr-d_5

Figure S35. HSQC spectrum of oxychiliotriterpenoside E (**5**) in Pyr-d_5

Figure S36. HMBC spectrum of oxychiliotriterpenosideE (**5**) in Pyr-d_5

Figure S37. NOESY spectrum of oxychiliotriterpenoside E (**5**) in Pyr-d_5

Figure S38. HRESIMS spectrum of oxychiliotriterpenoside E 6'-methyl ester (**6**)

Figure S39. ^{1}H NMR spectrum of oxychiliotriterpenoside E 6'-methyl ester (**6**) in Pyr-d_5

Figure S40. ^{13}C NMR spectrum of oxychiliotriterpenoside E 6'-methyl ester (**6**) in Pyr-d_5

Figure S41. ^{1}H-^{1}H COSY spectrum of oxychiliotriterpenoside E 6'-methyl ester (**6**) in Pyr-d_5

Figure S42. HSQC spectrum of oxychiliotriterpenoside E 6'-methyl ester (**6**) in Pyr-d_5

Figure S43. HMBC spectrum of oxychiliotriterpenoside E 6'-methyl ester (**6**) in Pyr-d_5

Figure S44. HRESIMS spectrum of myrioside B 6'-methyl ester (**7**)

Figure S45. ^{1}H NMR spectrum of myrioside B 6'- methyl ester (**7**) in Pyr-d_5

Figure S46. ^{13}C NMR spectrum of myrioside B 6'-methyl ester (**7**) in Pyr-d_5

Figure S47. ^{1}H-^{1}H COSY spectrum of myrioside B 6'-methyl ester (**7**) in Pyr-d_5

Figure S48. HSQC spectrum of myrioside B 6'- methyl ester (**7**) in Pyr-d_5

Figure S49. HMBC spectrum of myrioside B 6'-methyl ester (**7**) in Pyr-d_5

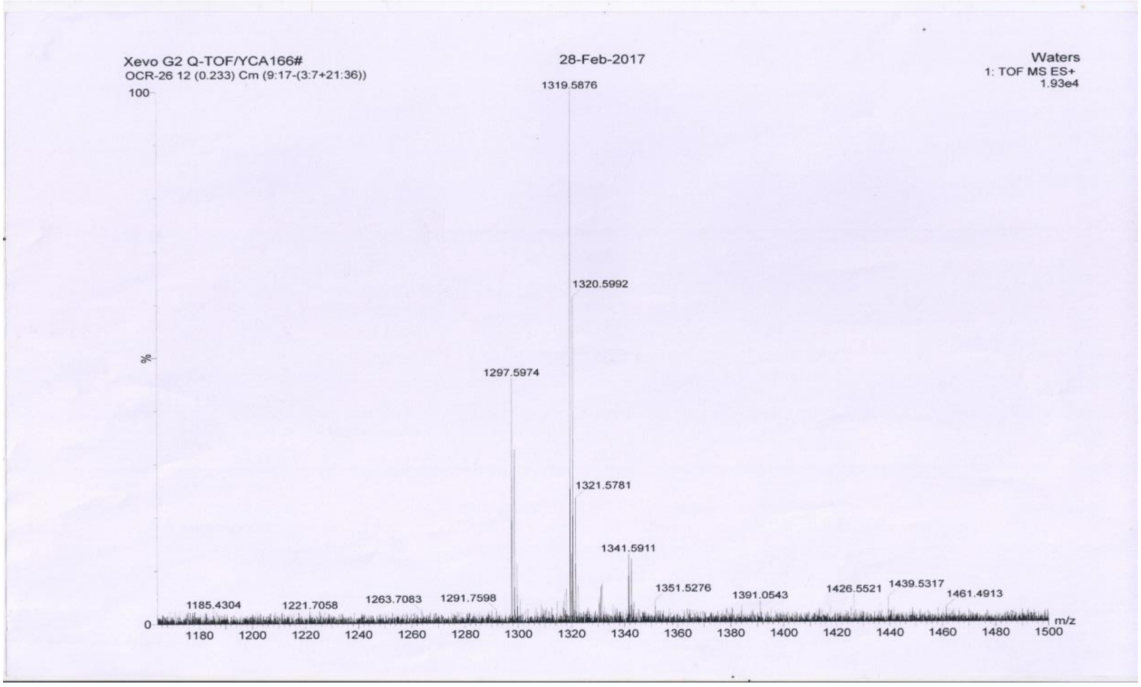

Figure S1. HRESIMS spectrum of oxychiliotriterpenoside A (**1**)

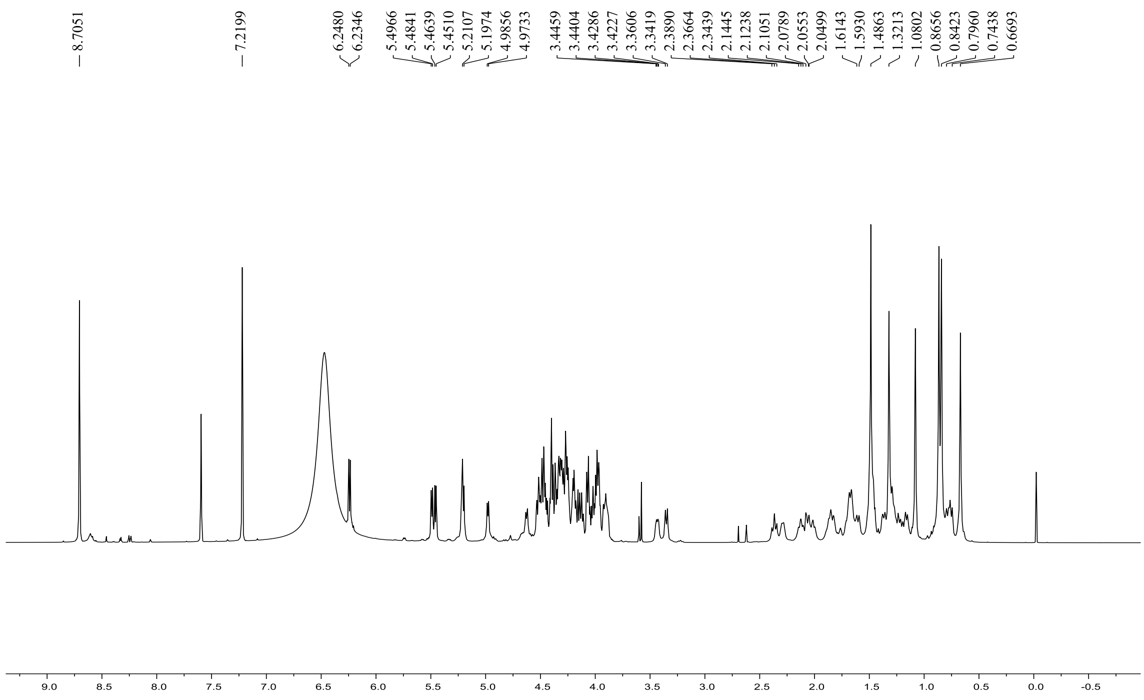

Figure S2. ^{1}H NMR spectrum of oxychiliotriterpenoside A (**1**) in Pyr-d_5

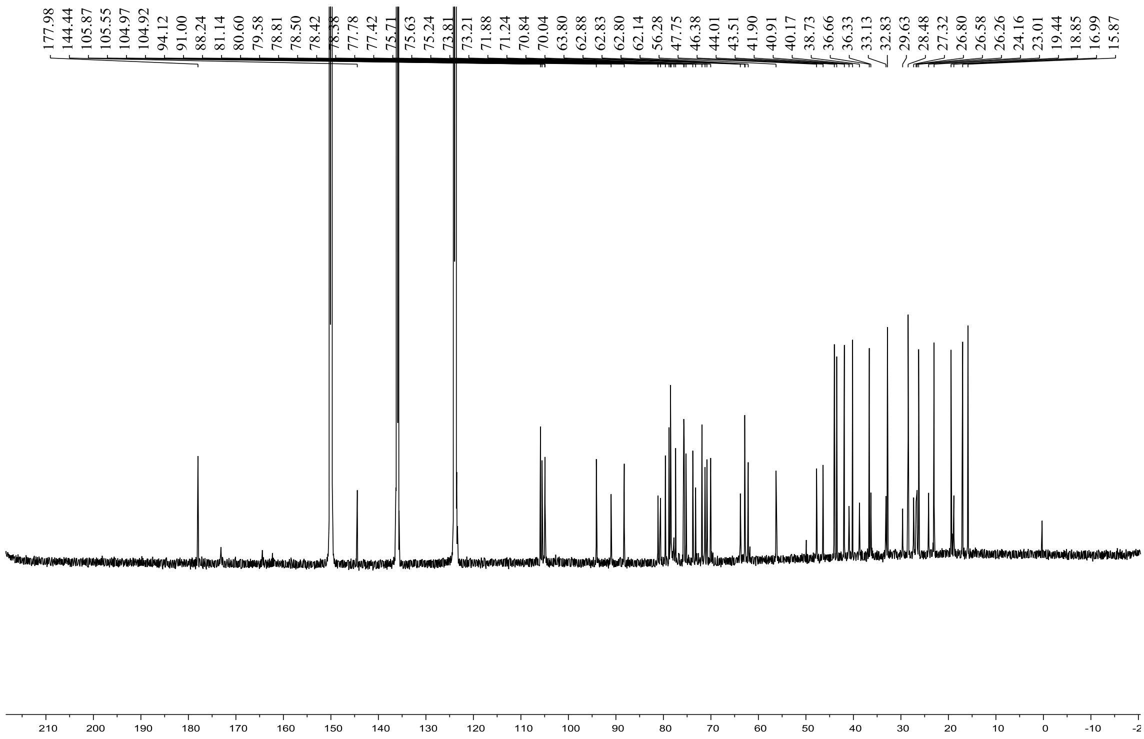

Figure S3. ^{13}C NMR spectrum of oxychiliotriterpenoside A (**1**) in Pyr-d_5

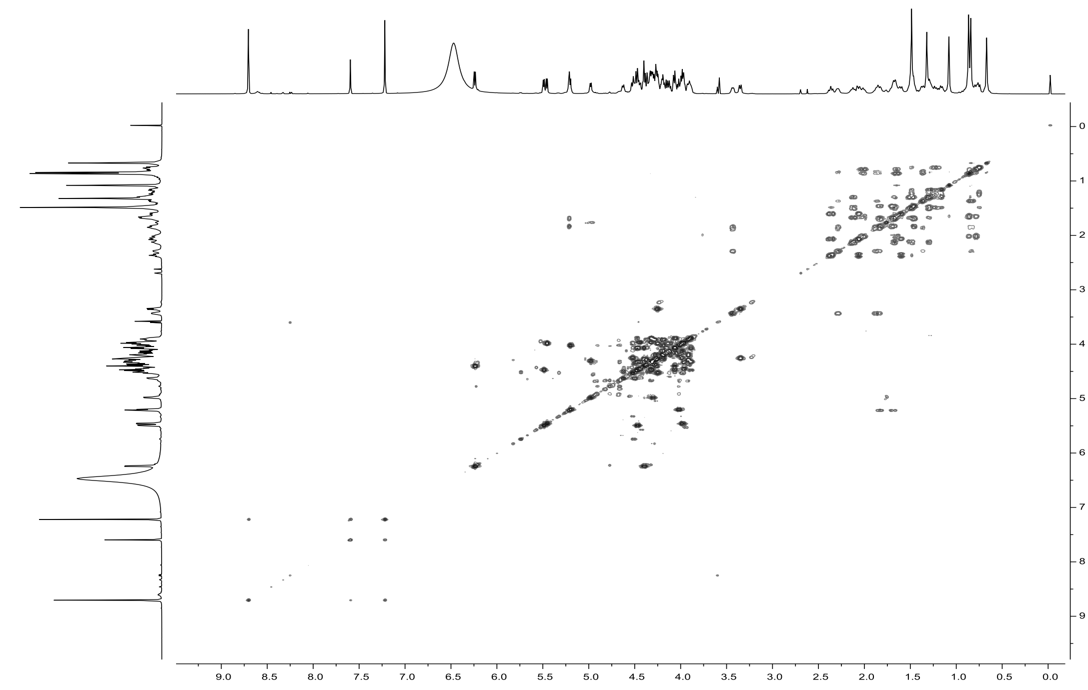

Figure S4. ^{1}H-^{1}H COSY spectrum of oxychiliotriterpenoside A (**1**) in Pyr-d_5

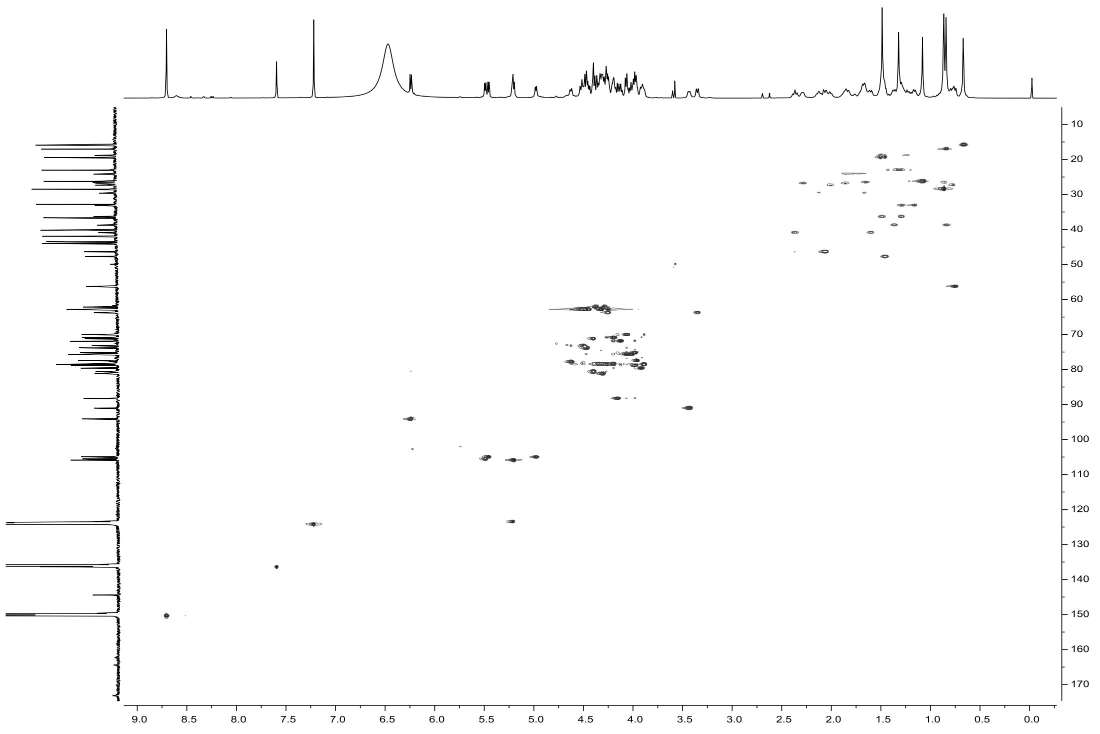

Figure S5. HSQC spectrum of oxychiliotriterpenoside A (**1**) in Pyr-d_5

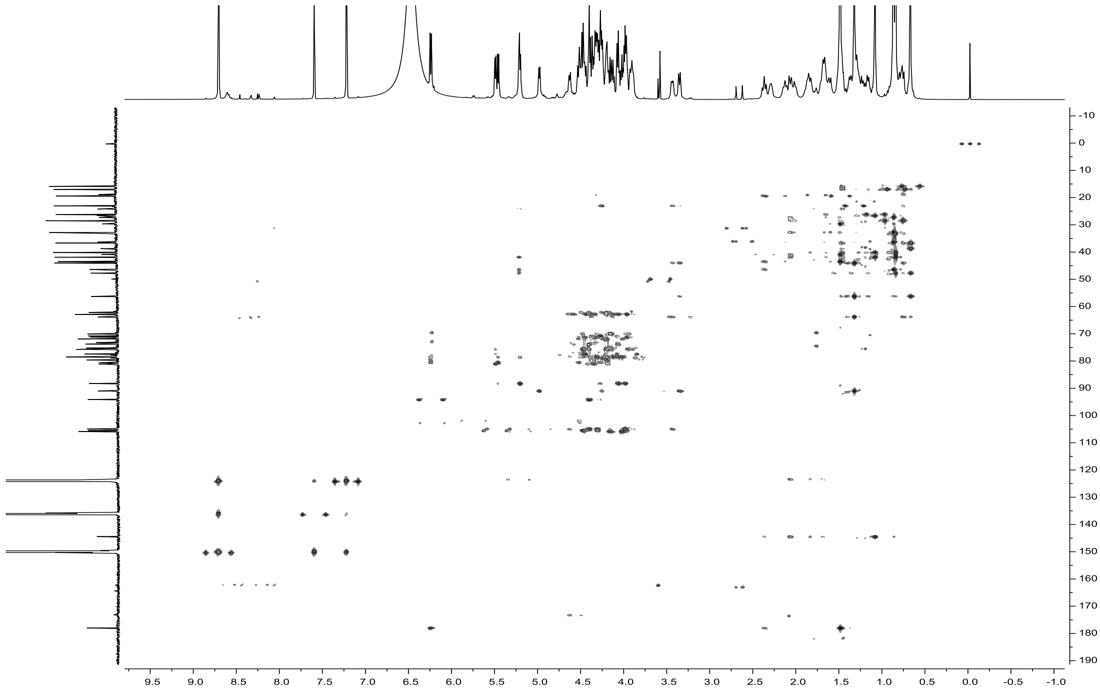

Figure S6. HMBC spectrum of oxychiliotriterpenoside A (**1**) in Pyr-d_5

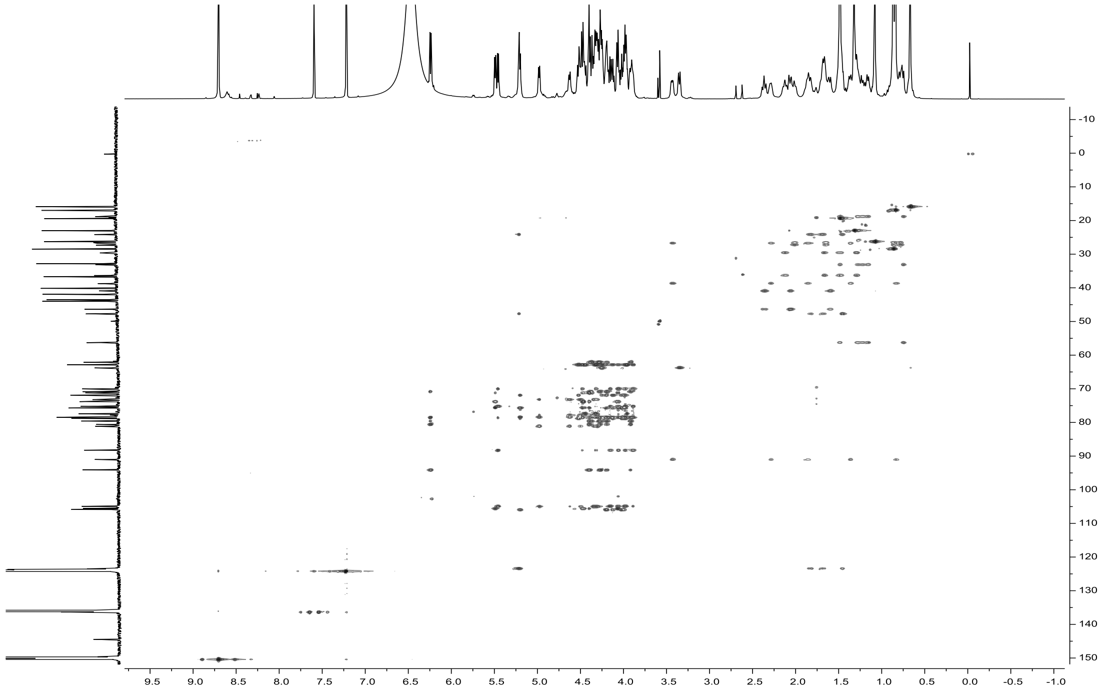

Figure S7. HSQC-TOCSY spectrum of oxychiliotriterpenoside A (**1**)in Pyr-d_5

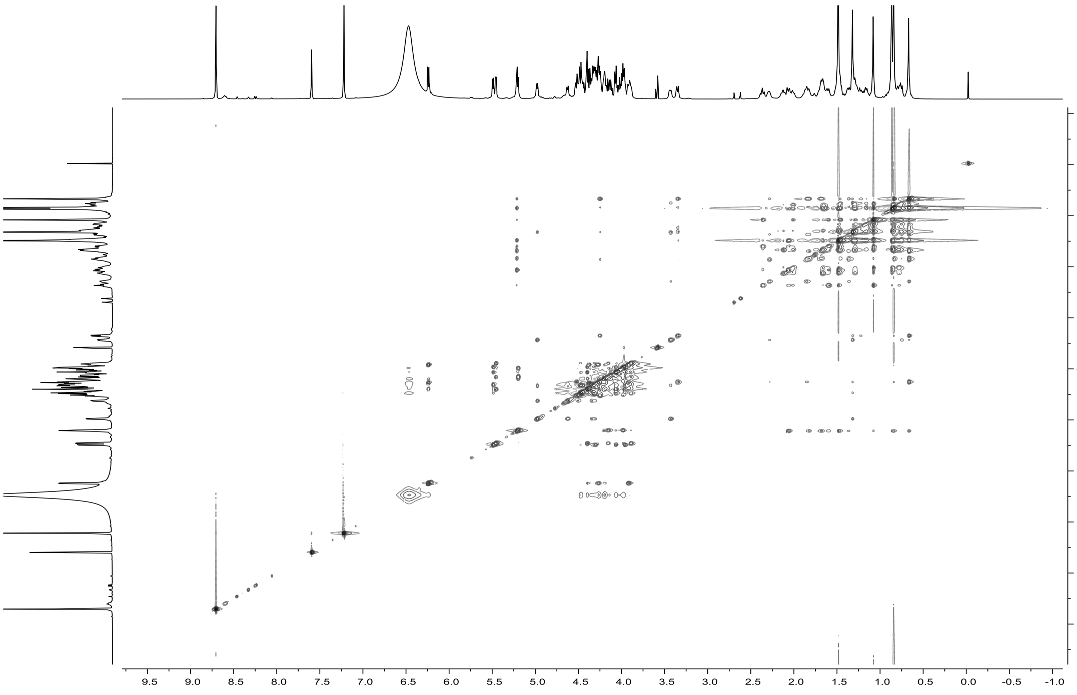

Figure S8. NOESY spectrum of oxychiliotriterpenoside A (**1**)in Pyr-*d*$_5$

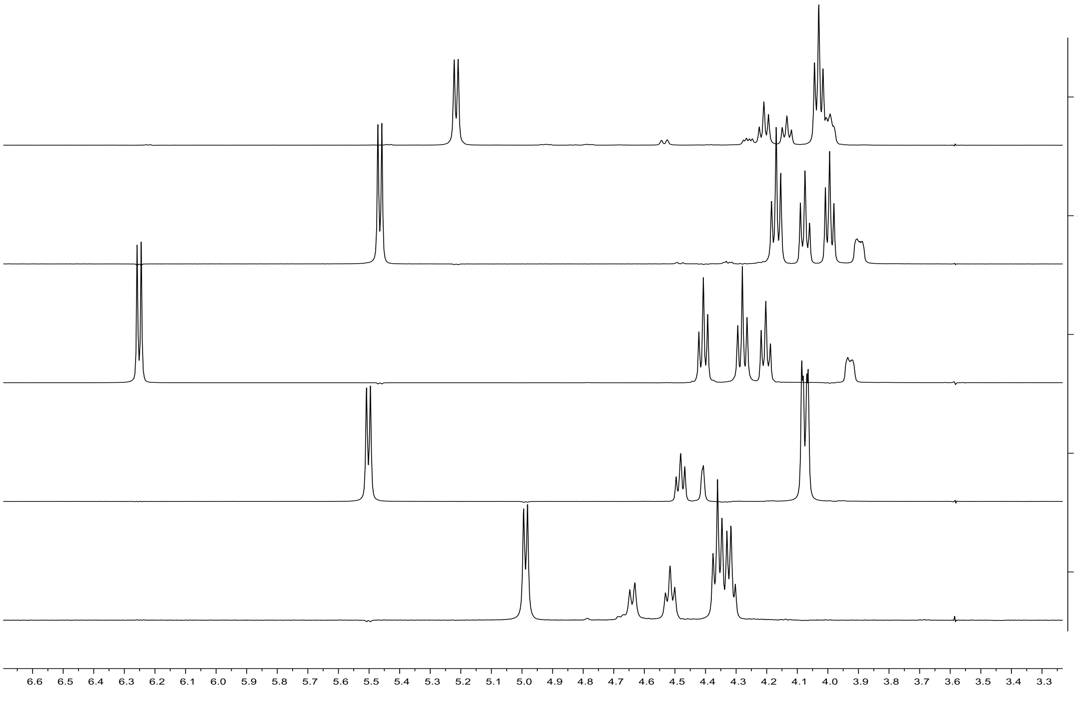

Figure S9. Selected 1D TOCSY spectrum of oxychiliotriterpenoside A (**1**)in Pyr-*d*$_5$

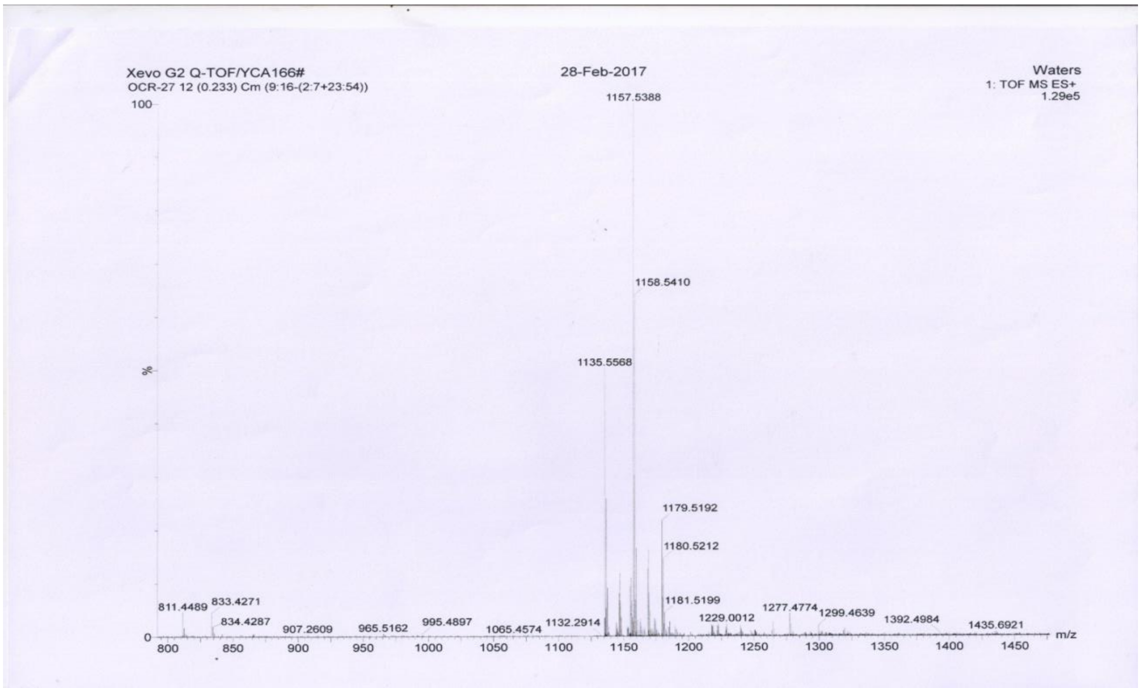

Figure S10. HRESIMS spectrum of oxychiliotriterpenoside B (**2**)

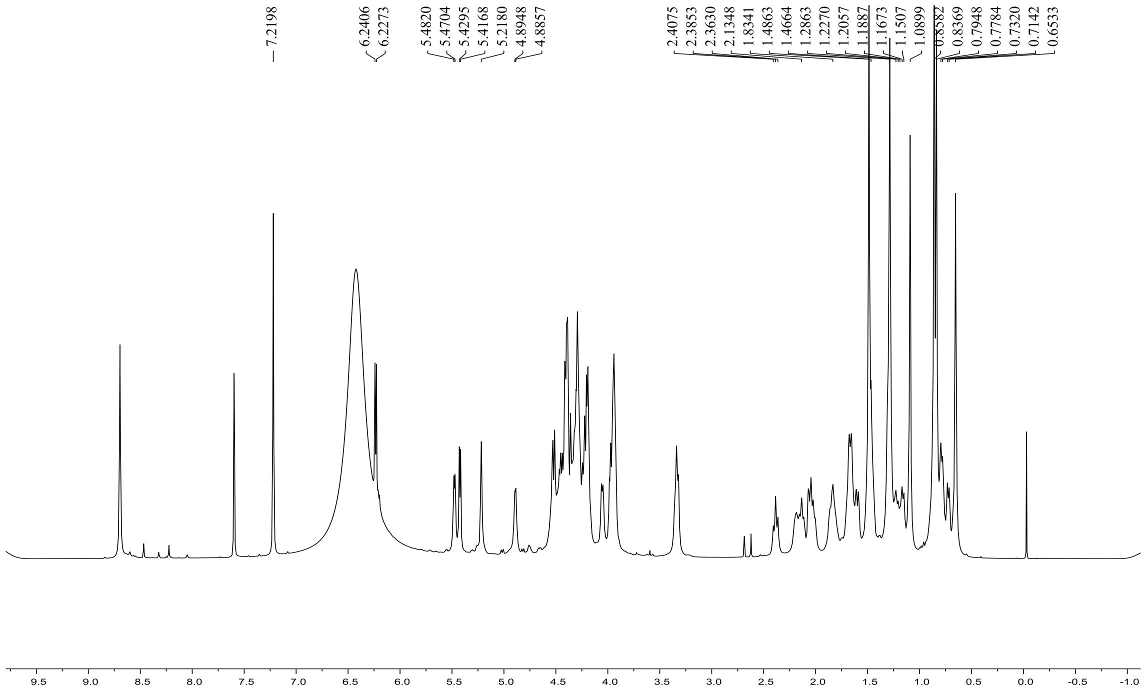

Figure S11. ^{1}H NMR spectrum of oxychiliotriterpenoside B (**2**) in Pyr-*d*$_5$

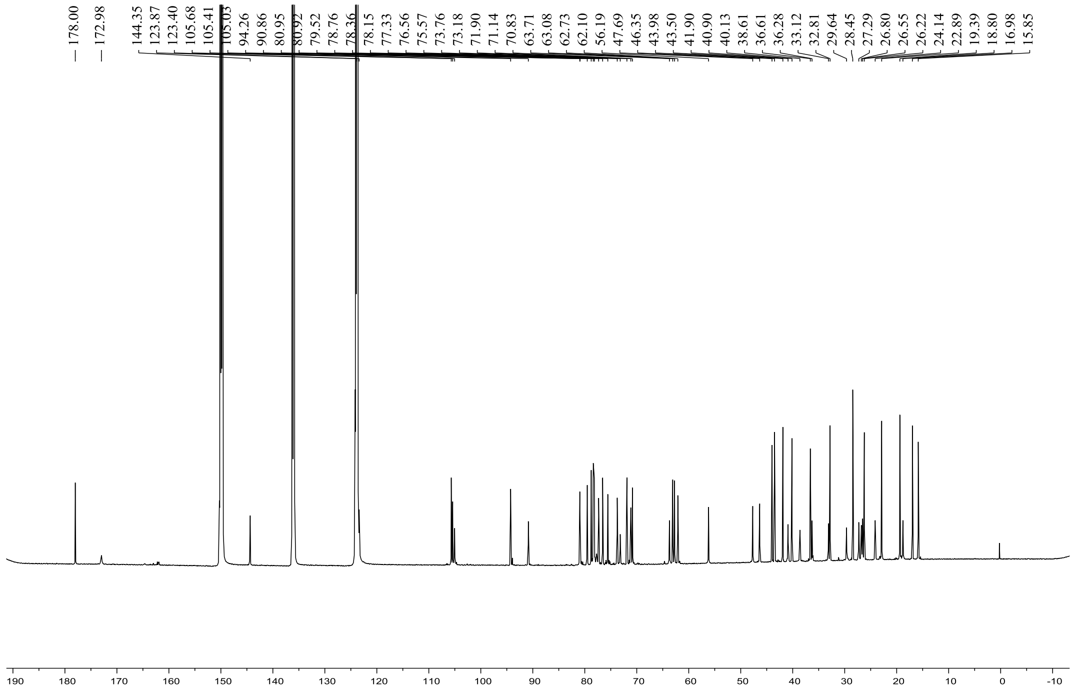

Figure S12. ^{13}C NMR spectrum of oxychiliotriterpenoside B (**2**) in Pyr-*d*$_5$

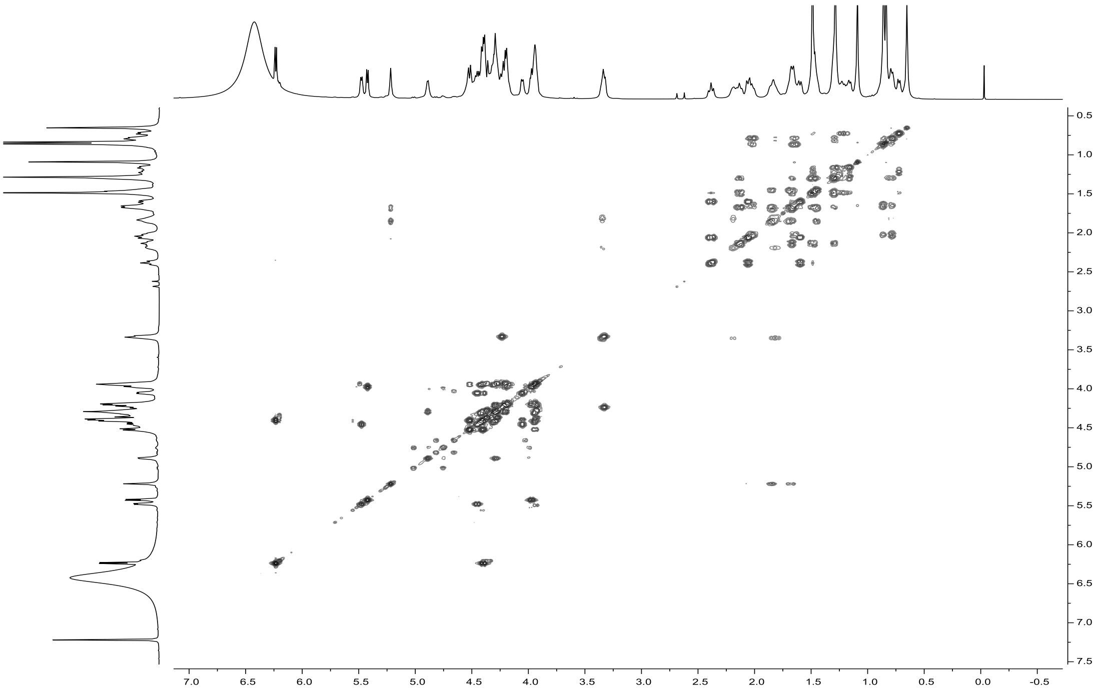

Figure S13. ^{1}H-^{1}H COSY spectrum of oxychiliotriterpenoside B (**2**) in Pyr-d_5

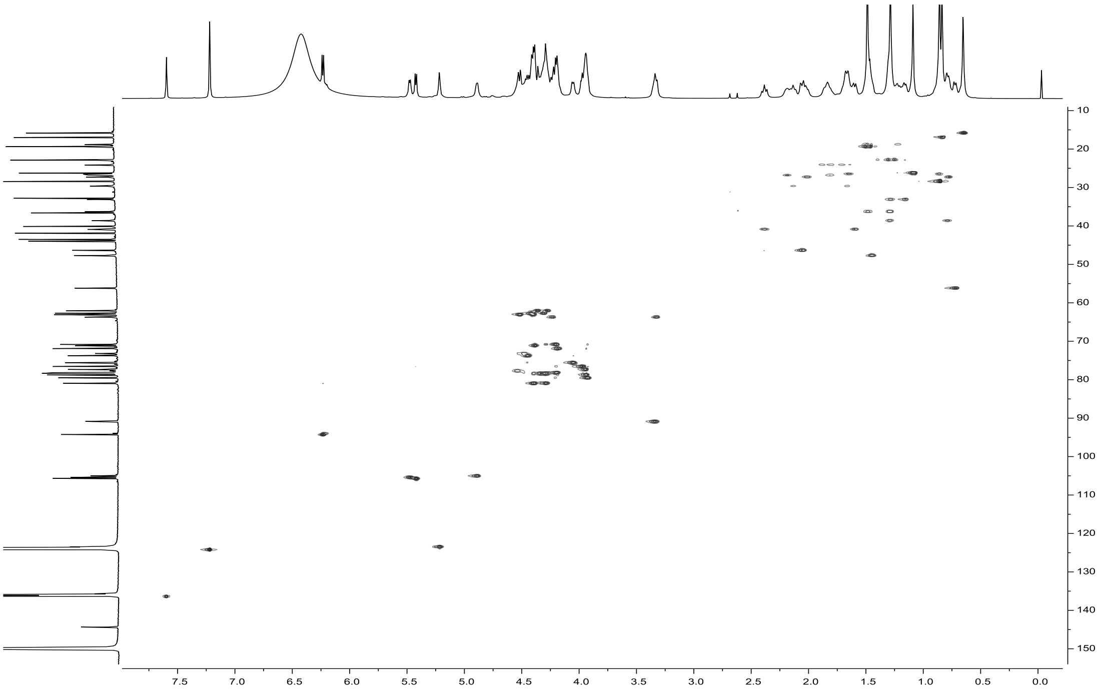

Figure S14. HSQC spectrum of oxychiliotriterpenoside B (**2**) in Pyr-d_5

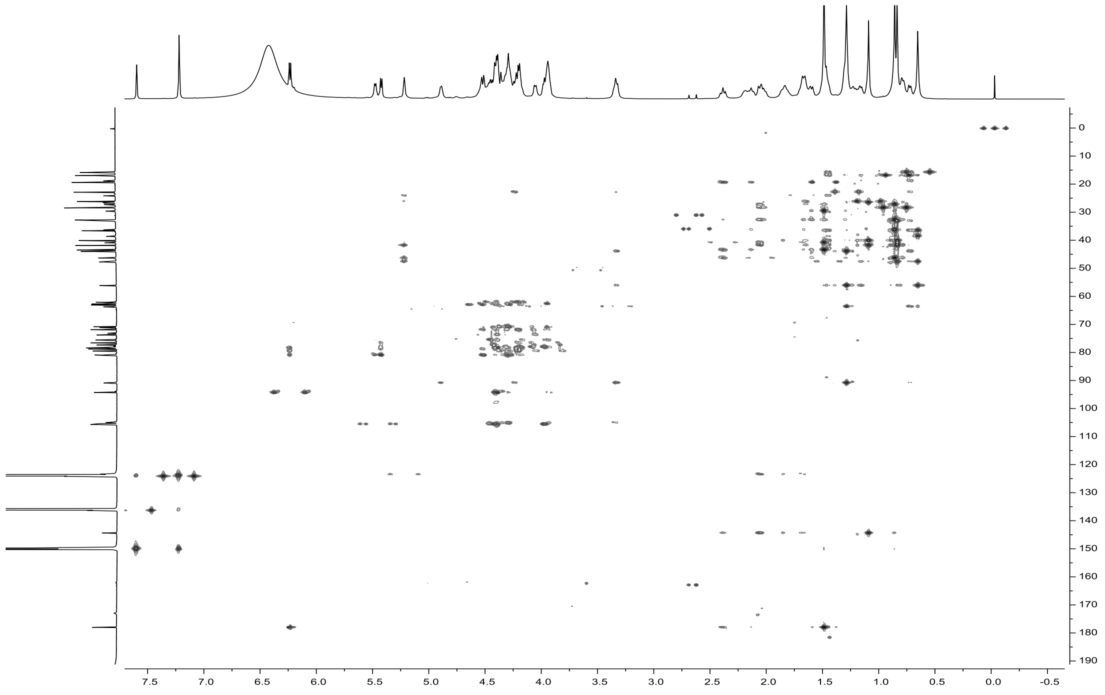

Figure S15. HMBC spectrum of oxychiliotriterpenoside B (**2**) in Pyr-d_5

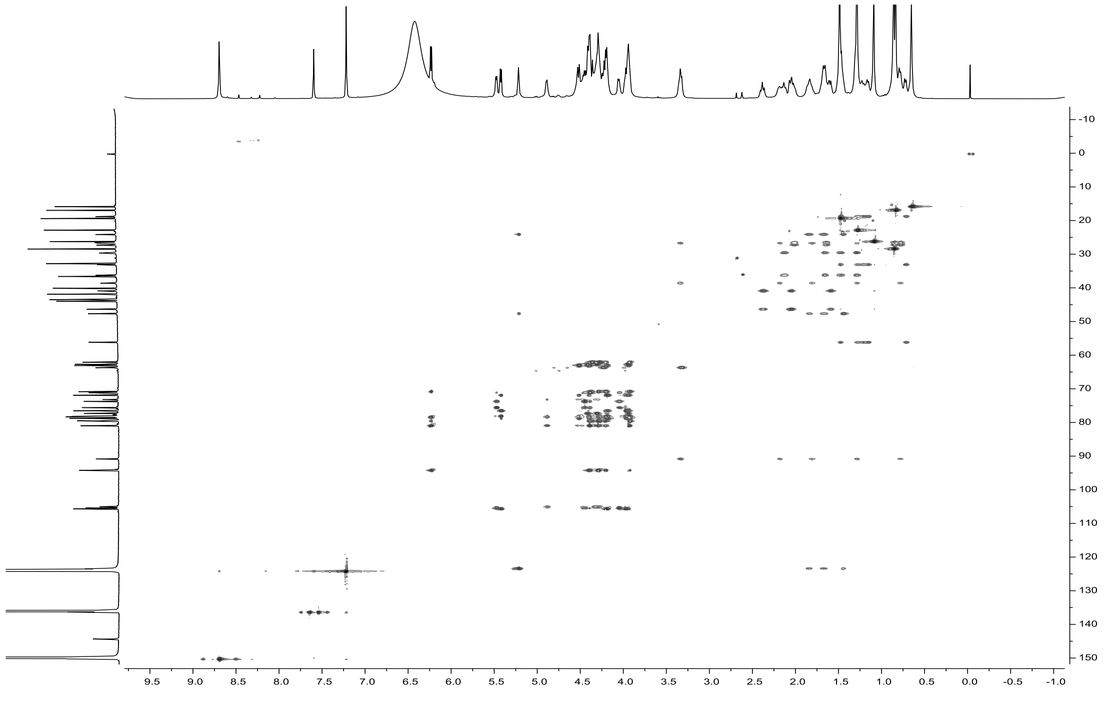

Figure S16. HSQC-TOCSY spectrum of oxychiliotriterpenoside B (**2**)in Pyr-d_5

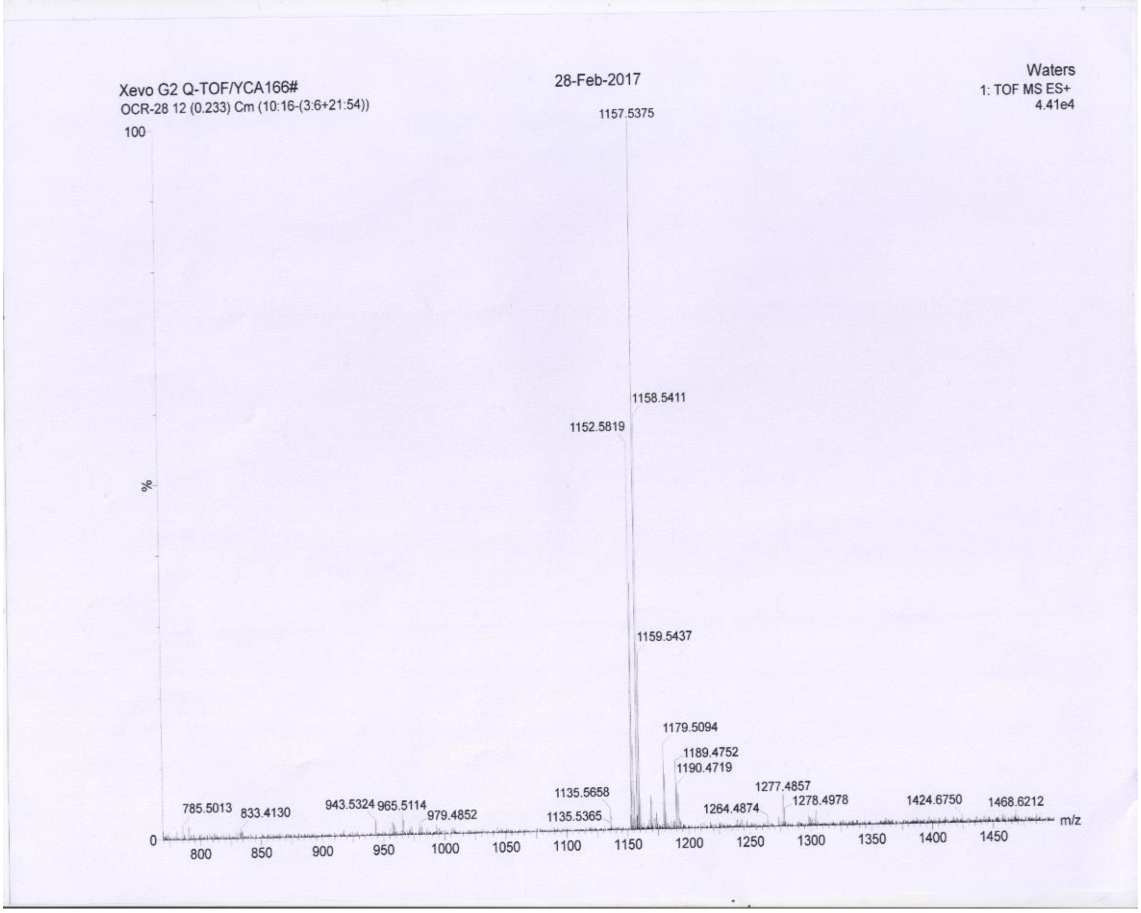

Figure S17. HRESIMS spectrum of oxychiliotriterpenoside C (**3**)

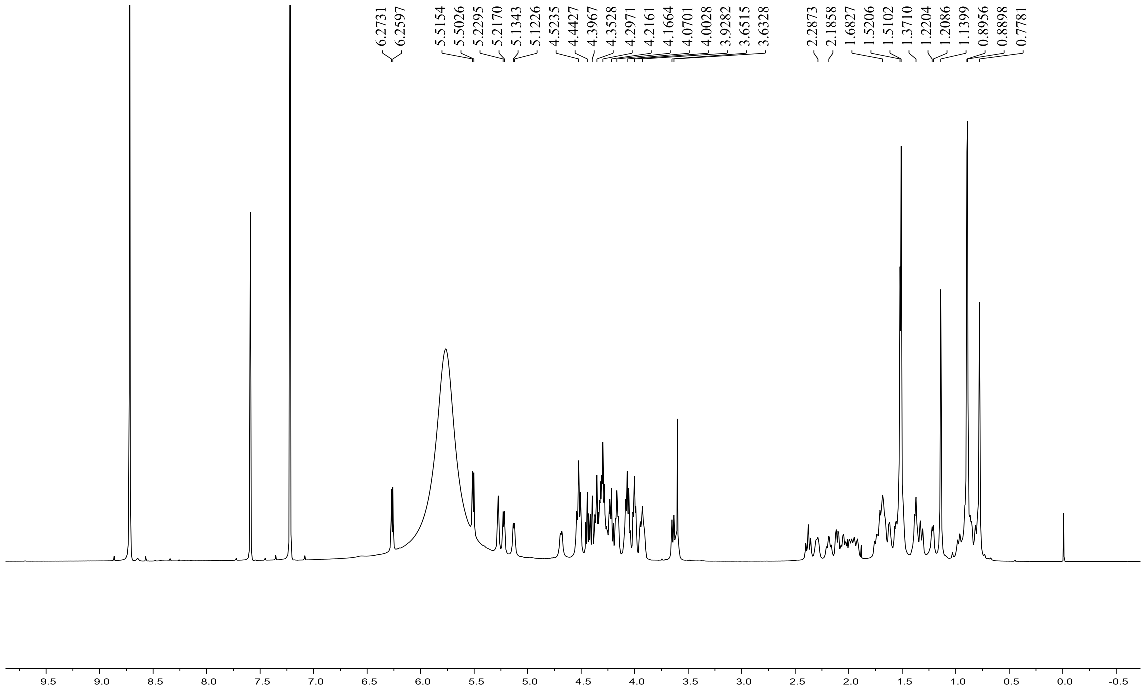

Figure S18. ^{1}H NMR spectrum of oxychiliotriterpenoside C (**3**) in Pyr-d_5

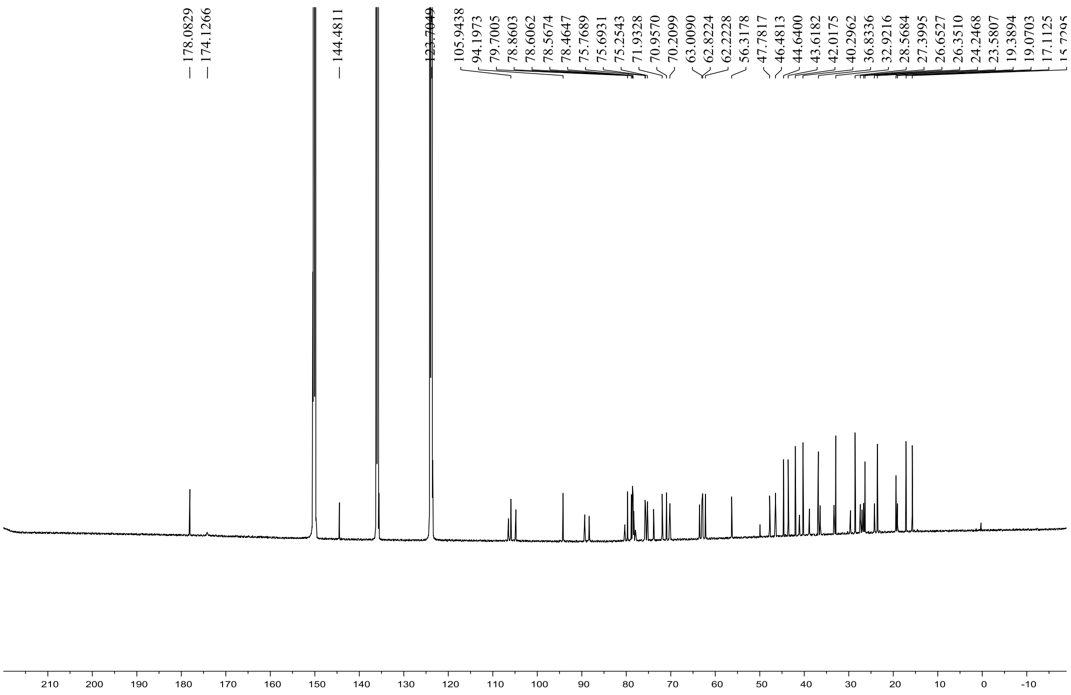

Figure S19. ^{13}C NMR spectrum of oxychiliotriterpenoside C (**3**) in Pyr-d_5

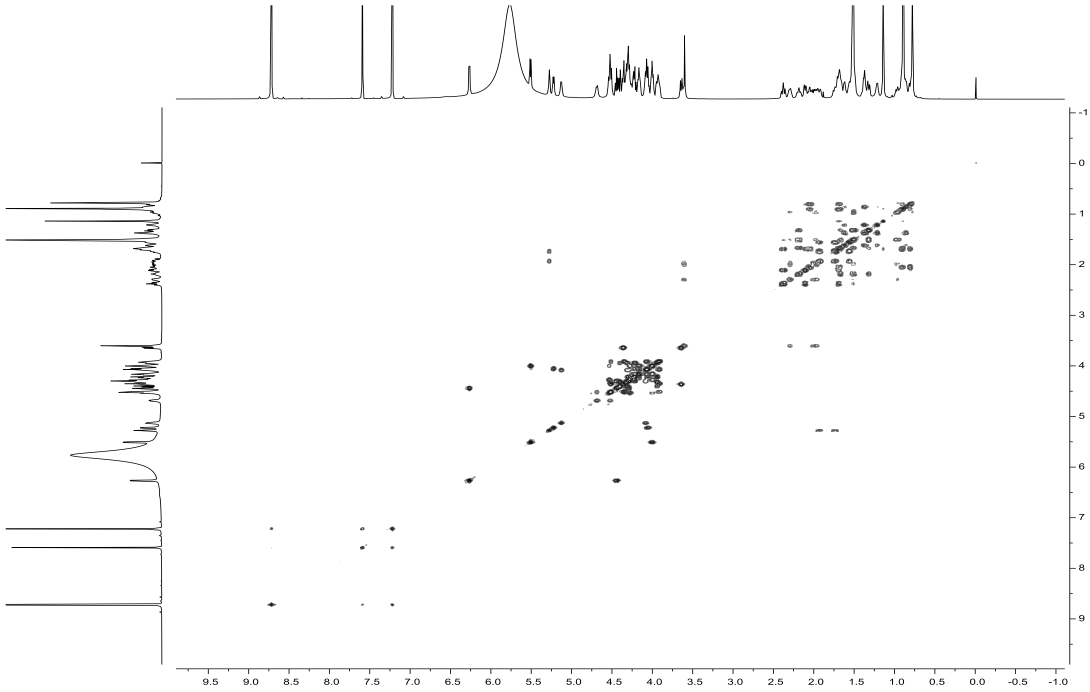

Figure S20. ^{1}H-^{1}H COSY spectrum of oxychiliotriterpenoside C (**3**) in Pyr-d_5

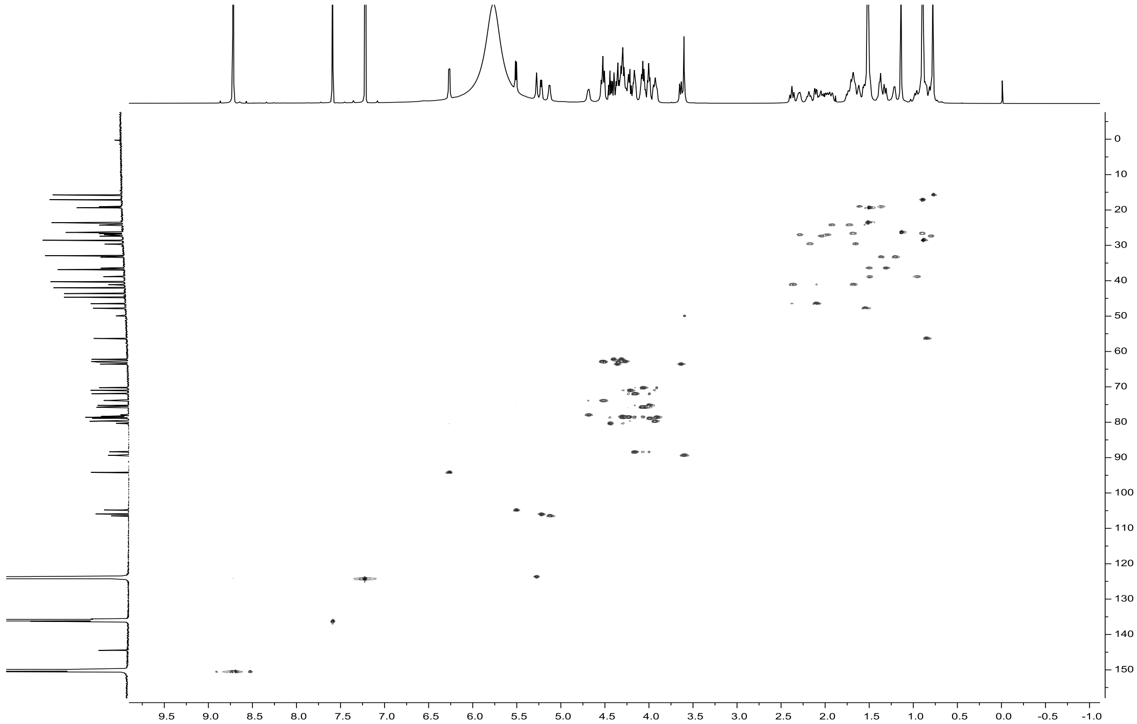

Figure S21. HSQC spectrum of oxychiliotriterpenoside C (**3**) in Pyr-d_5

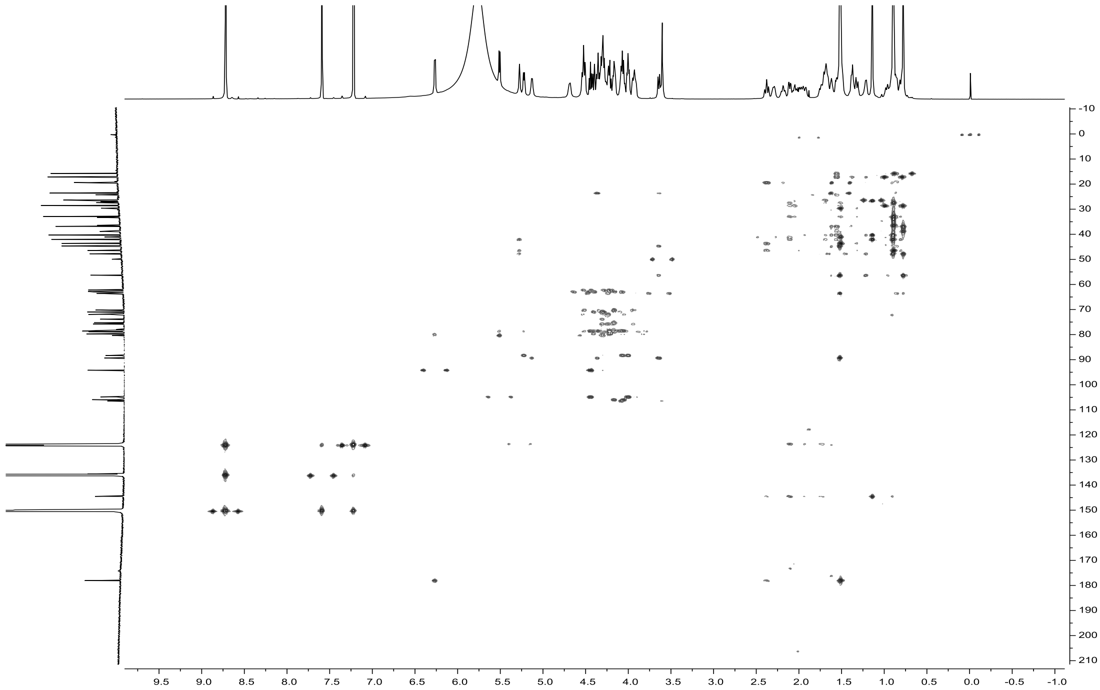

Figure S22. HMBC spectrum of oxychiliotriterpenosideC (**3**) in Pyr-d_5

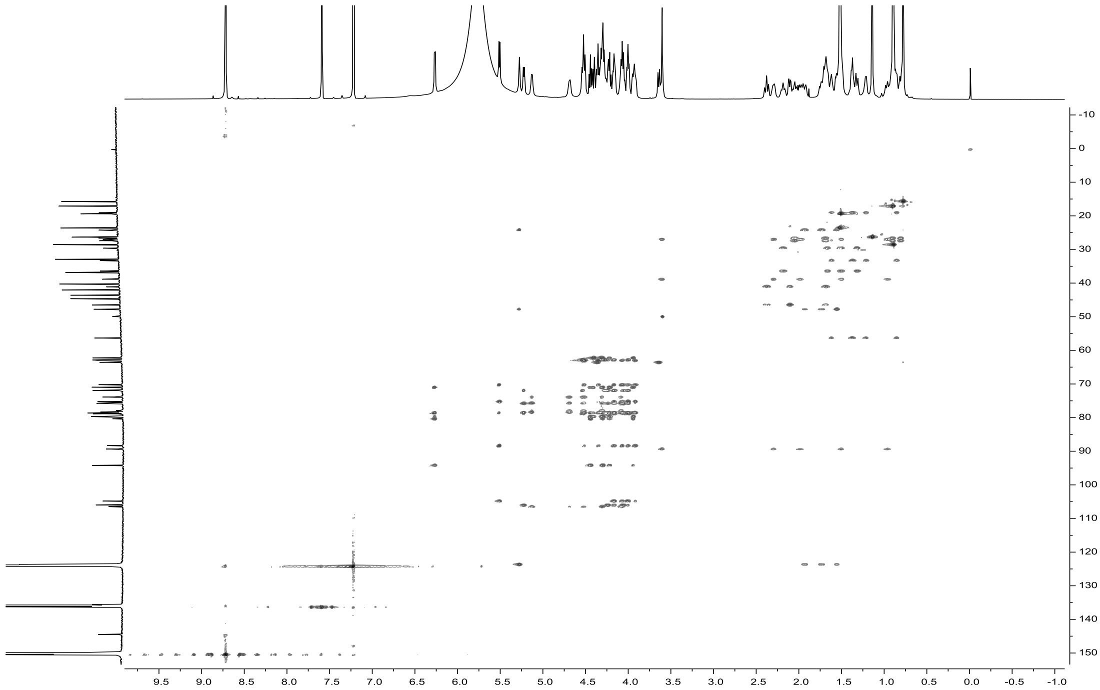

Figure S23. HSQC-TOCSY spectrum of oxychiliotriterpenoside C (**3**)in Pyr-d_5

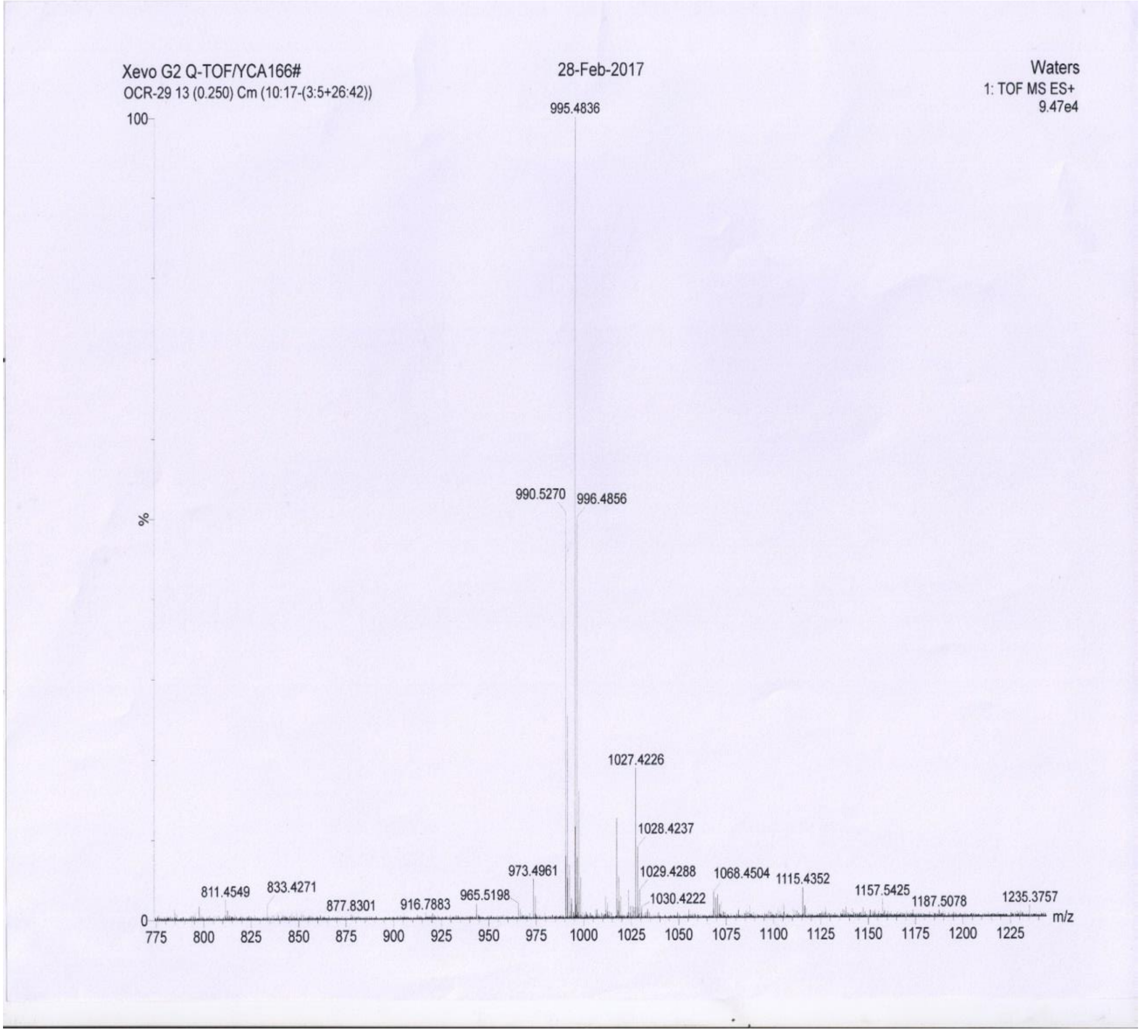

Figure S24. HRESIMS spectrum of oxychiliotriterpenoside D (**4**)

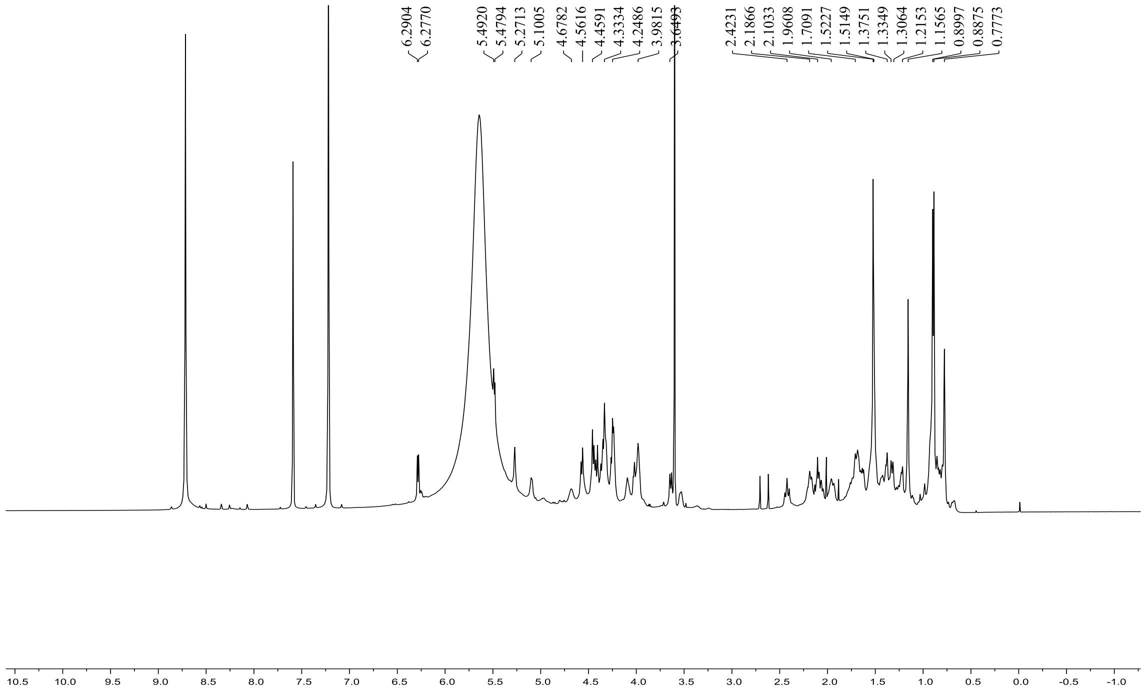

Figure S25. ^{1}H NMR spectrum of oxychiliotriterpenoside D (**4**) in Pyr-d_5

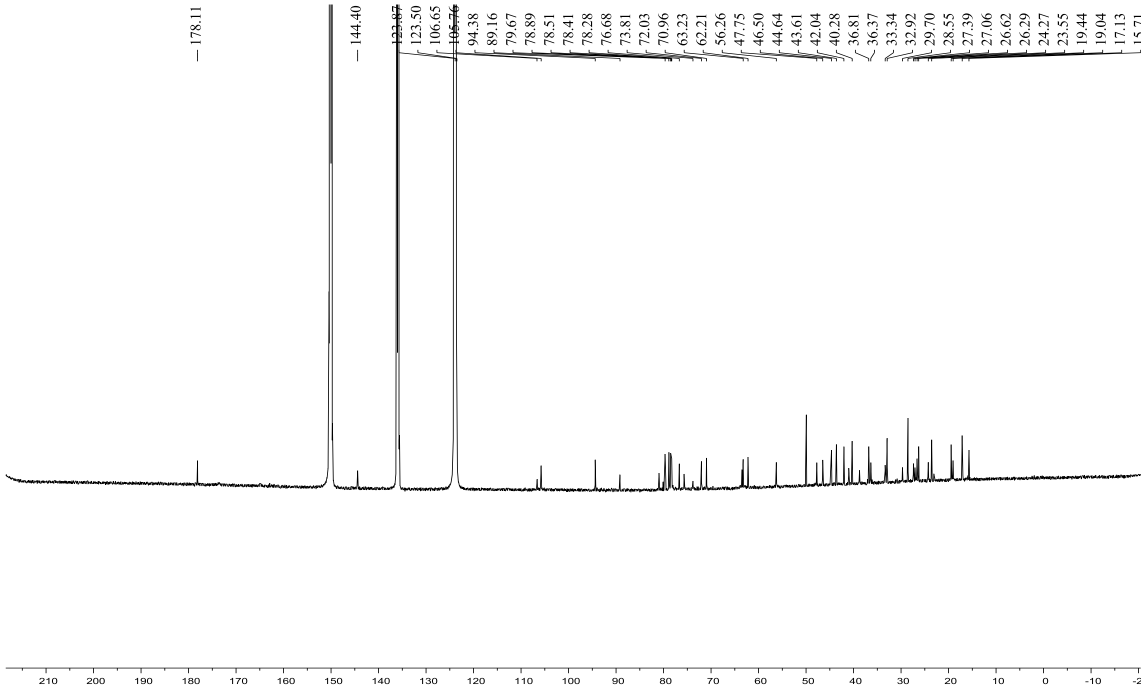

Figure S26. ^{13}C NMR spectrum of oxychiliotriterpenoside D (**4**) in Pyr-d_5

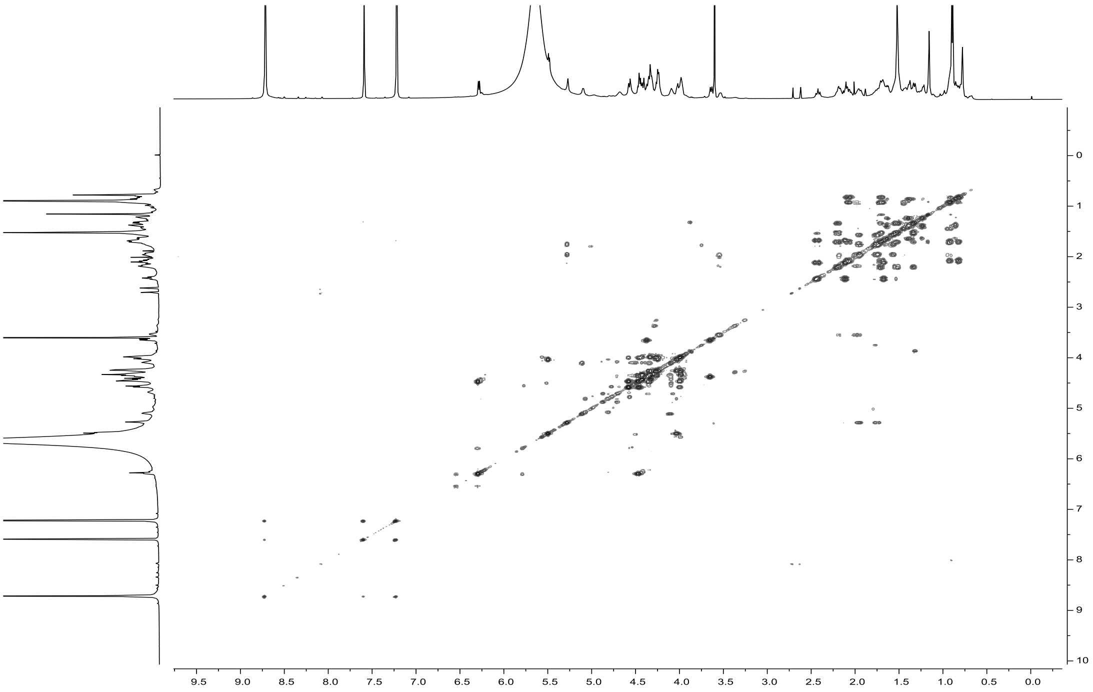

Figure S27. ^{1}H-^{1}H COSY spectrum of oxychiliotriterpenoside D (**4**) in Pyr-d_5

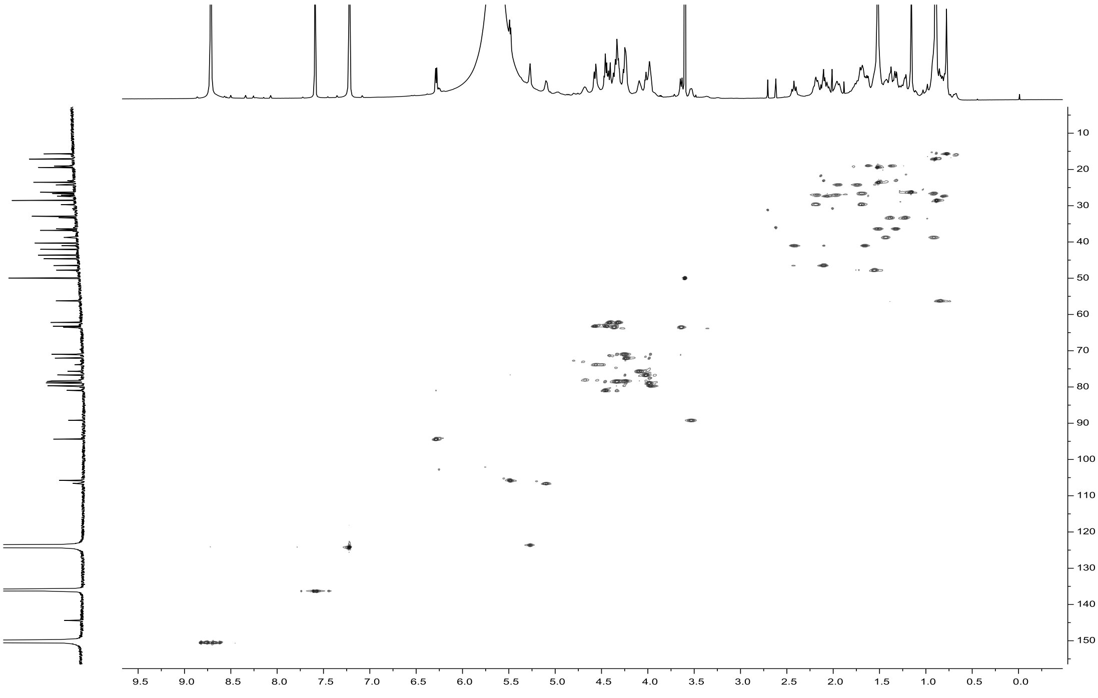

Figure S28. HSQC spectrum of oxychiliotriterpenoside D (**4**) in Pyr-d_5

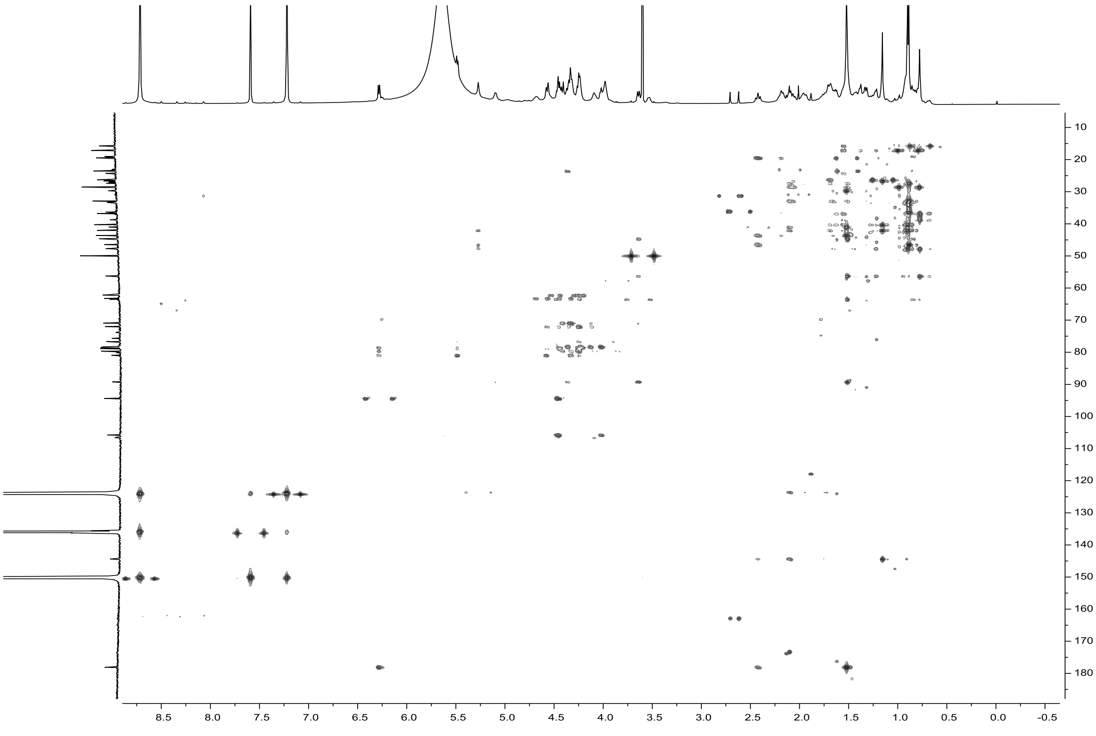

Figure S29. HMBC spectrum of oxychiliotriterpenosideD (**4**) in Pyr-*d*$_5$

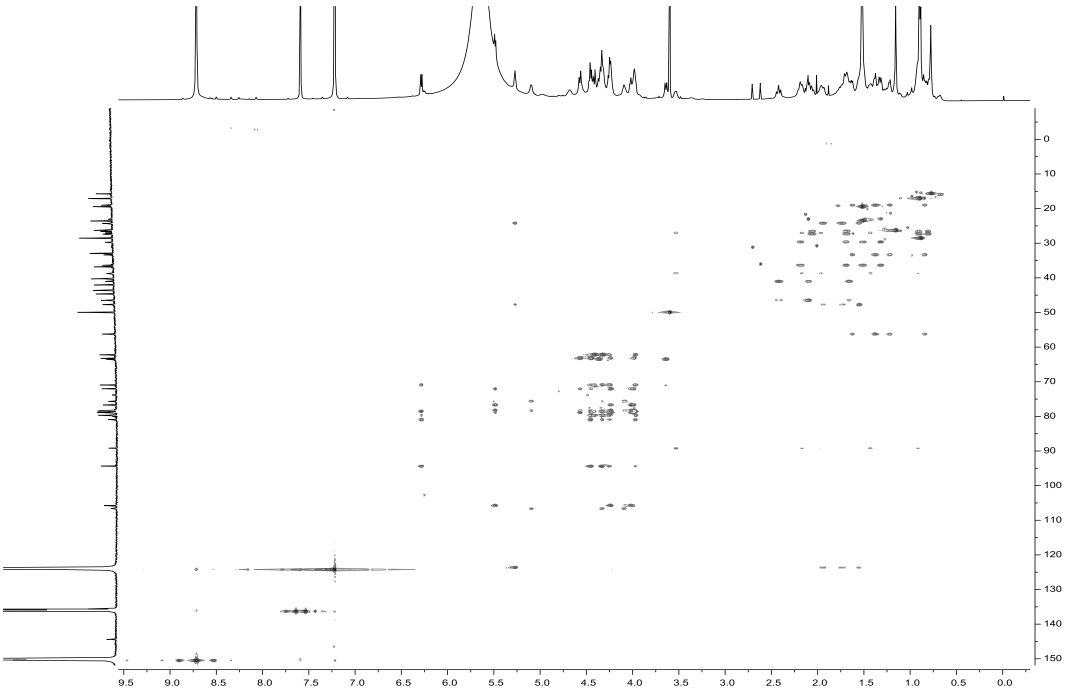

Figure S30. HSQC-TOCSY spectrum of oxychiliotriterpenoside D (**4**)in Pyr-*d*$_5$

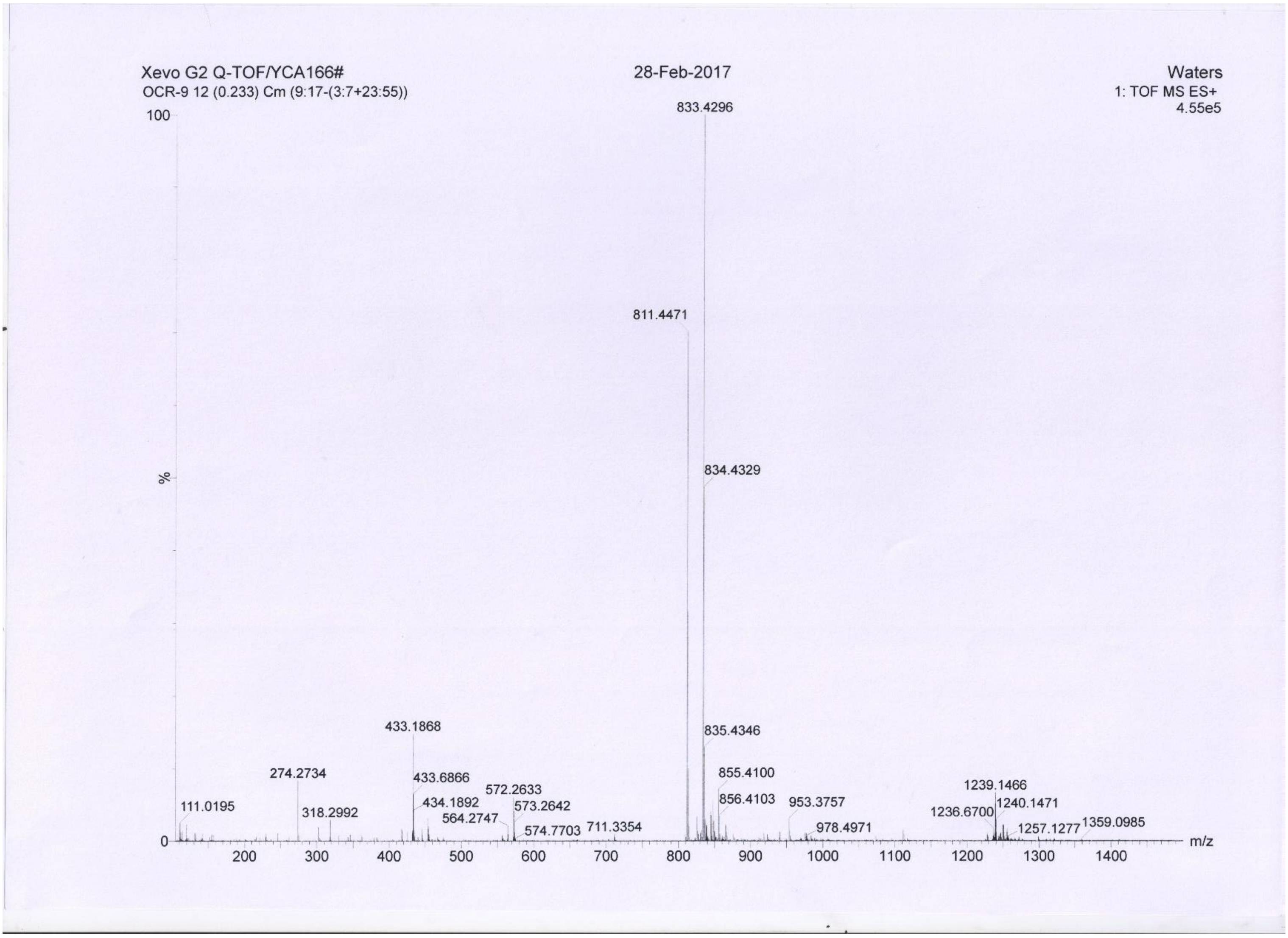

Figure S31. HRESIMS spectrum of oxychiliotriterpenoside E (**5**)

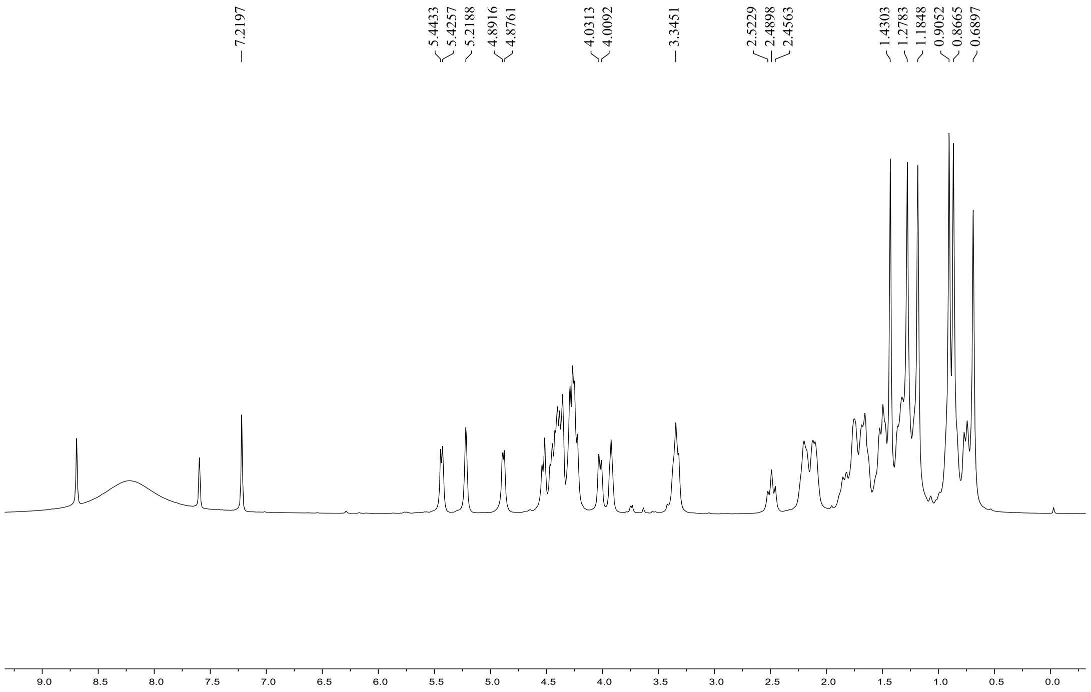

Figure S32. ^{1}H NMR spectrum of oxychiliotriterpenoside E (**5**) in Pyr-d_5

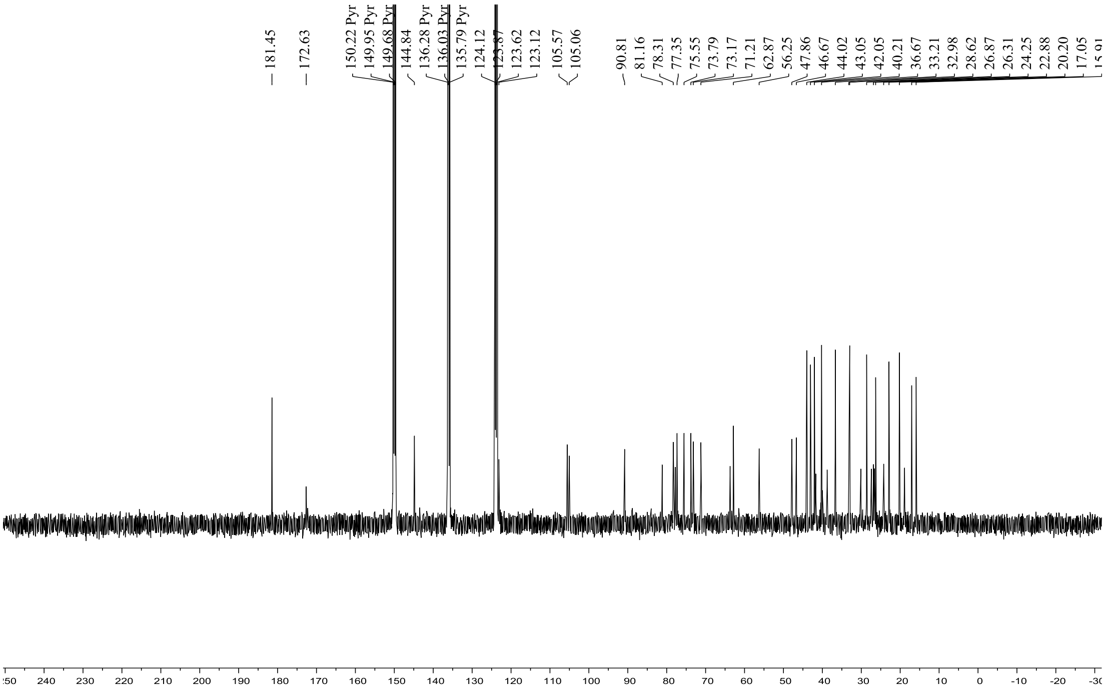

Figure S33. ^{13}C NMR spectrum of oxychiliotriterpenoside E (**5**) in Pyr-d_5

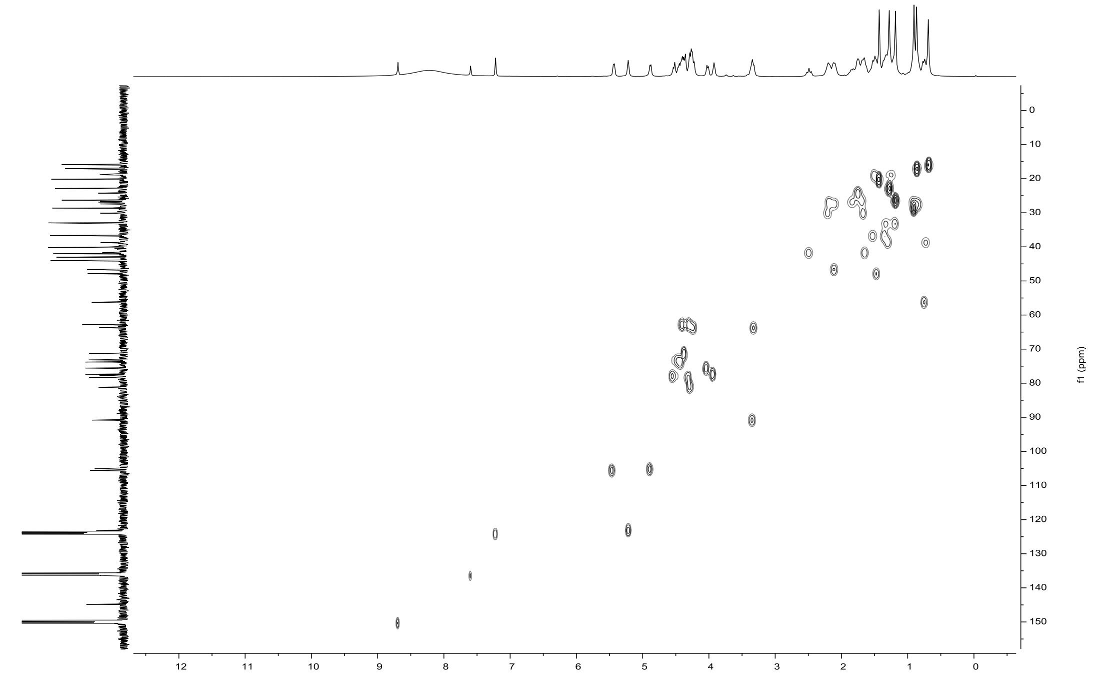

Figure S35. HSQC spectrum of oxychiliotriterpenoside E (**5**) in Pyr-d_5

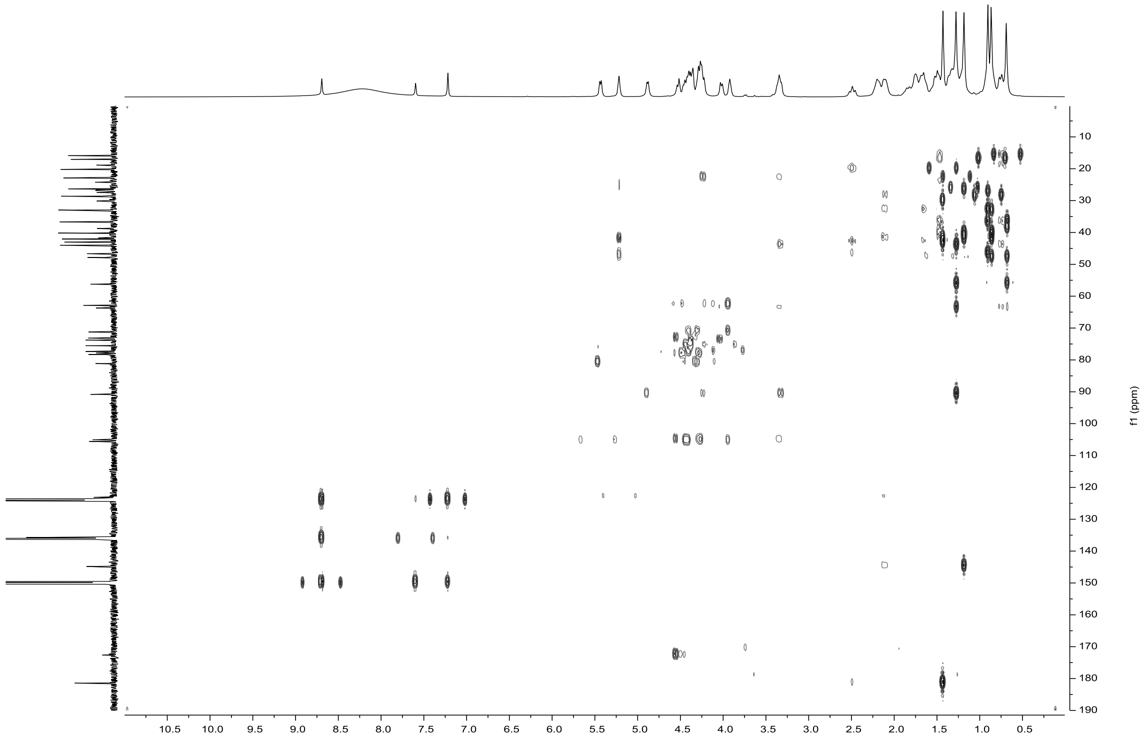

Figure S36. HMBC spectrum of oxychiliotriterpenosideE (**5**) in Pyr-d_5

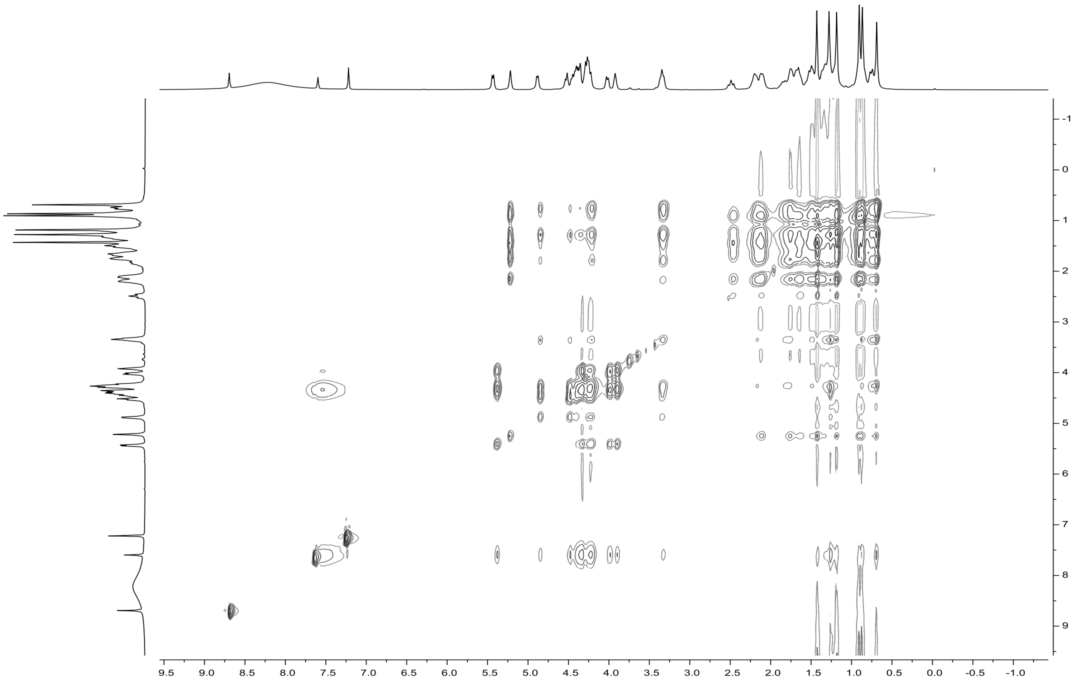

Figure S37. NOESY spectrum of oxychiliotriterpenoside E (**5**)in Pyr-d_5

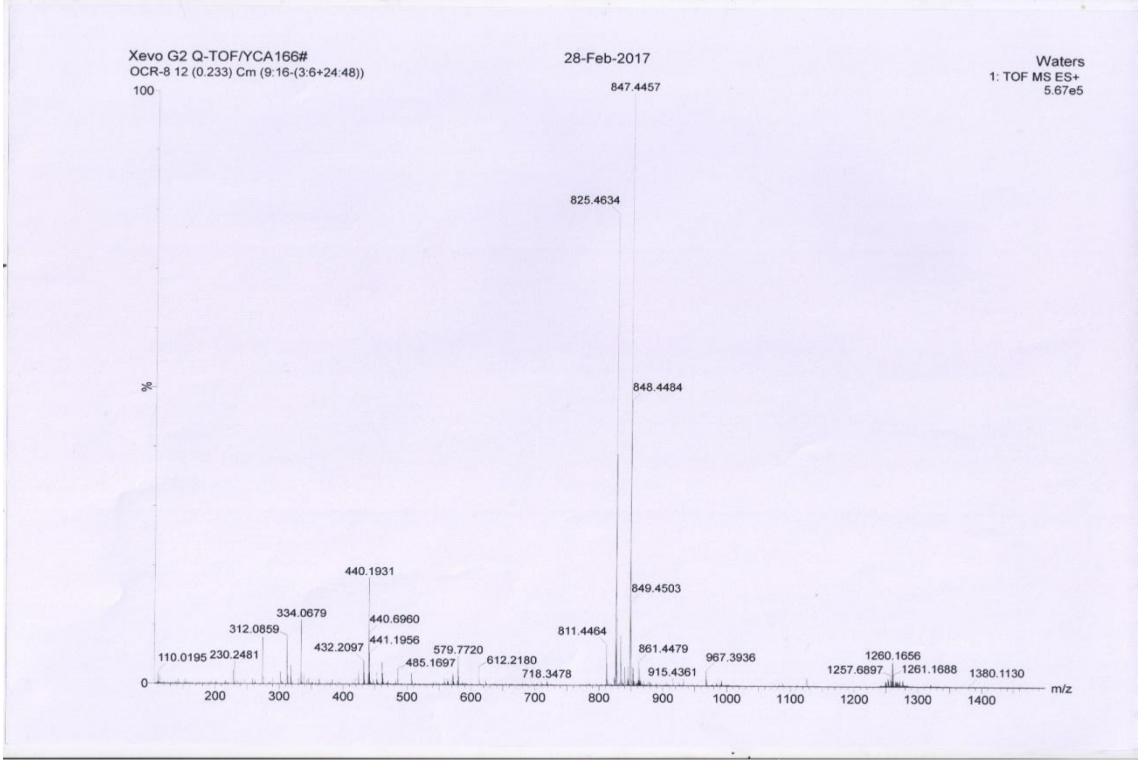

Figure S38. HRESIMS spectrum of oxychiliotriterpenoside E 6'-methyl ester (**6**)

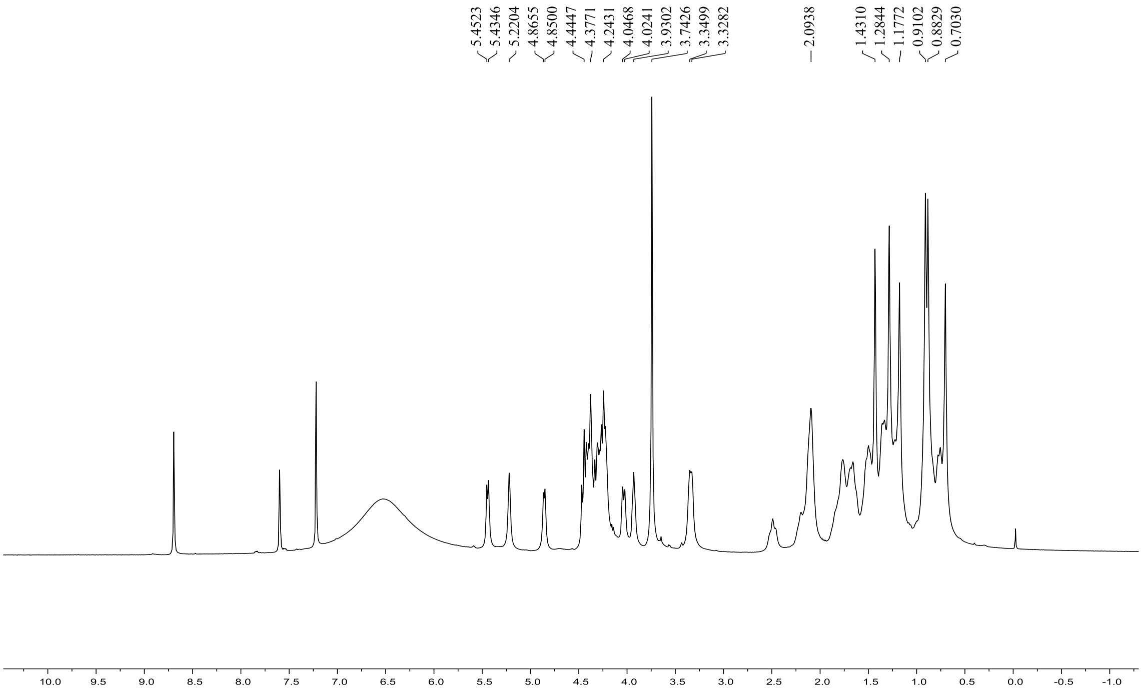

Figure S39. ^{1}H NMR spectrum of oxychiliotriterpenoside E 6'-methyl ester (**6**) in Pyr-d_5

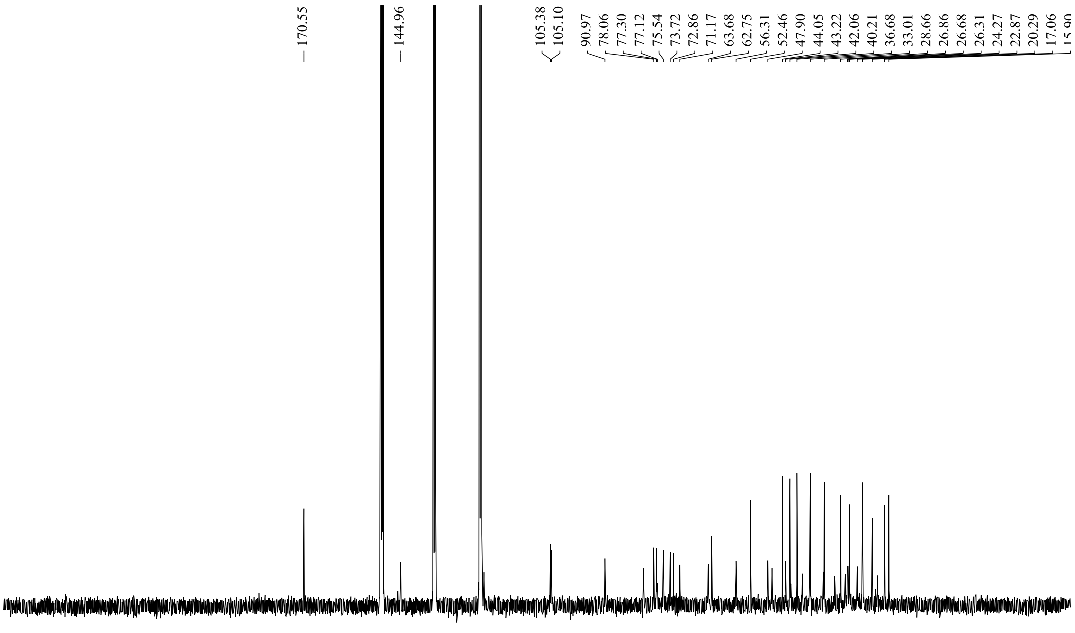

Figure S40. ^{13}C NMR spectrum of oxychiliotriterpenoside E 6'-methyl ester (**6**) in Pyr-d_5

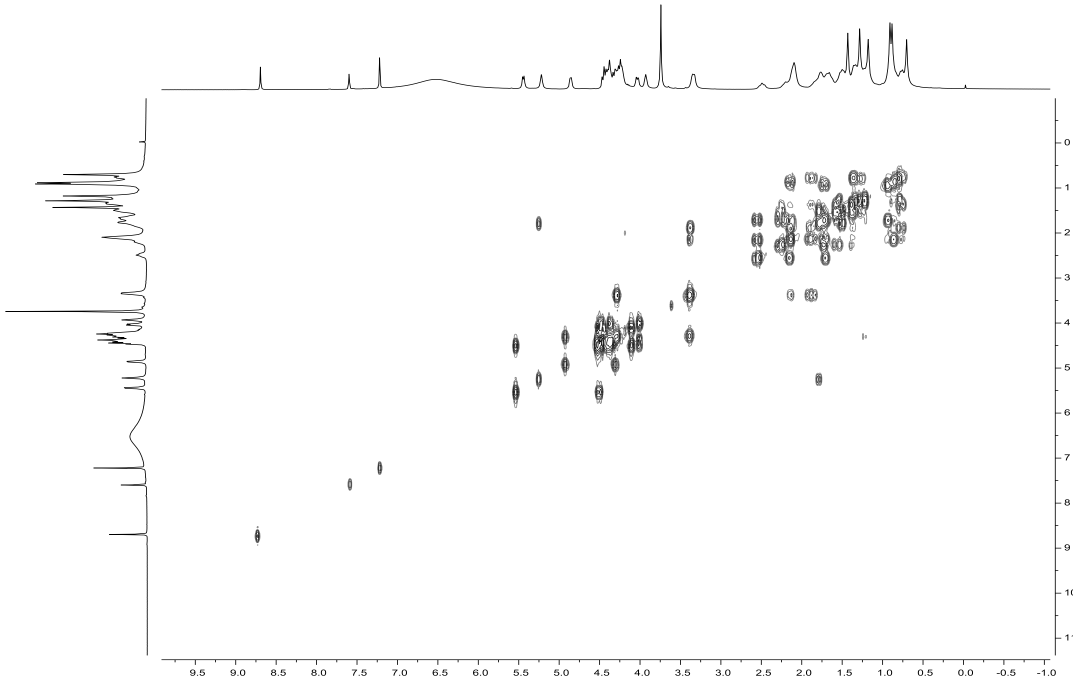

Figure S41. ^{1}H-^{1}H COSY spectrum of oxychiliotriterpenoside E 6'-methyl ester (**6**) in Pyr-d_5

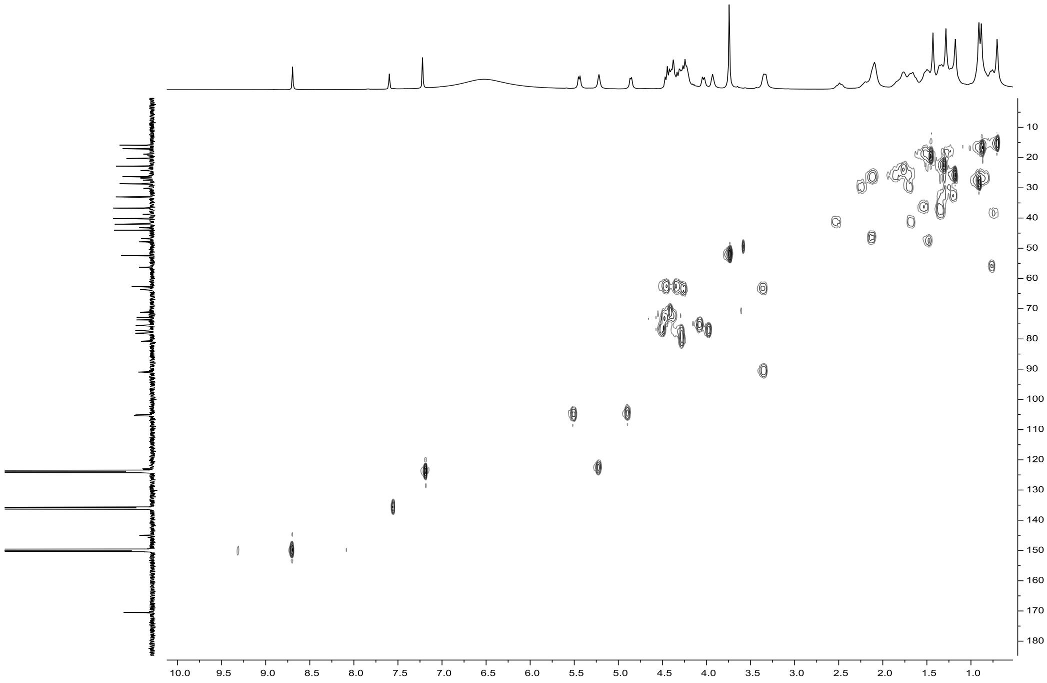

Figure S42. HSQC spectrum of oxychiliotriterpenoside E 6'-methyl ester (**6**) in Pyr-d_5

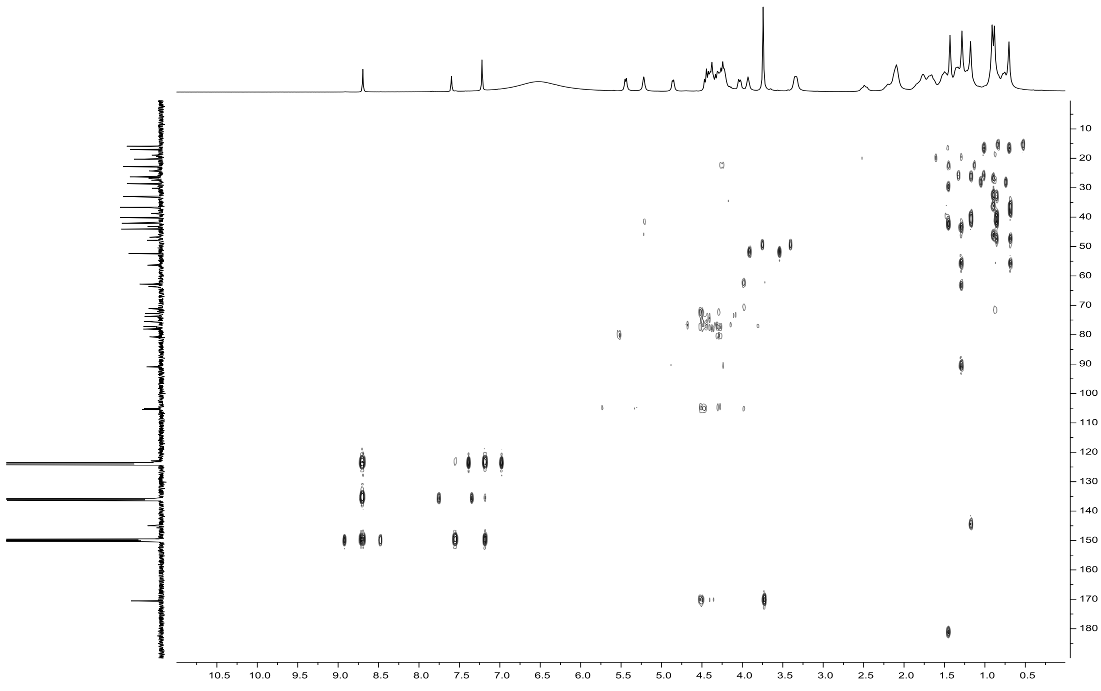

Figure S43. HMBC spectrum of oxychiliotriterpenoside E 6'-methyl ester (**6**) in Pyr-d_5

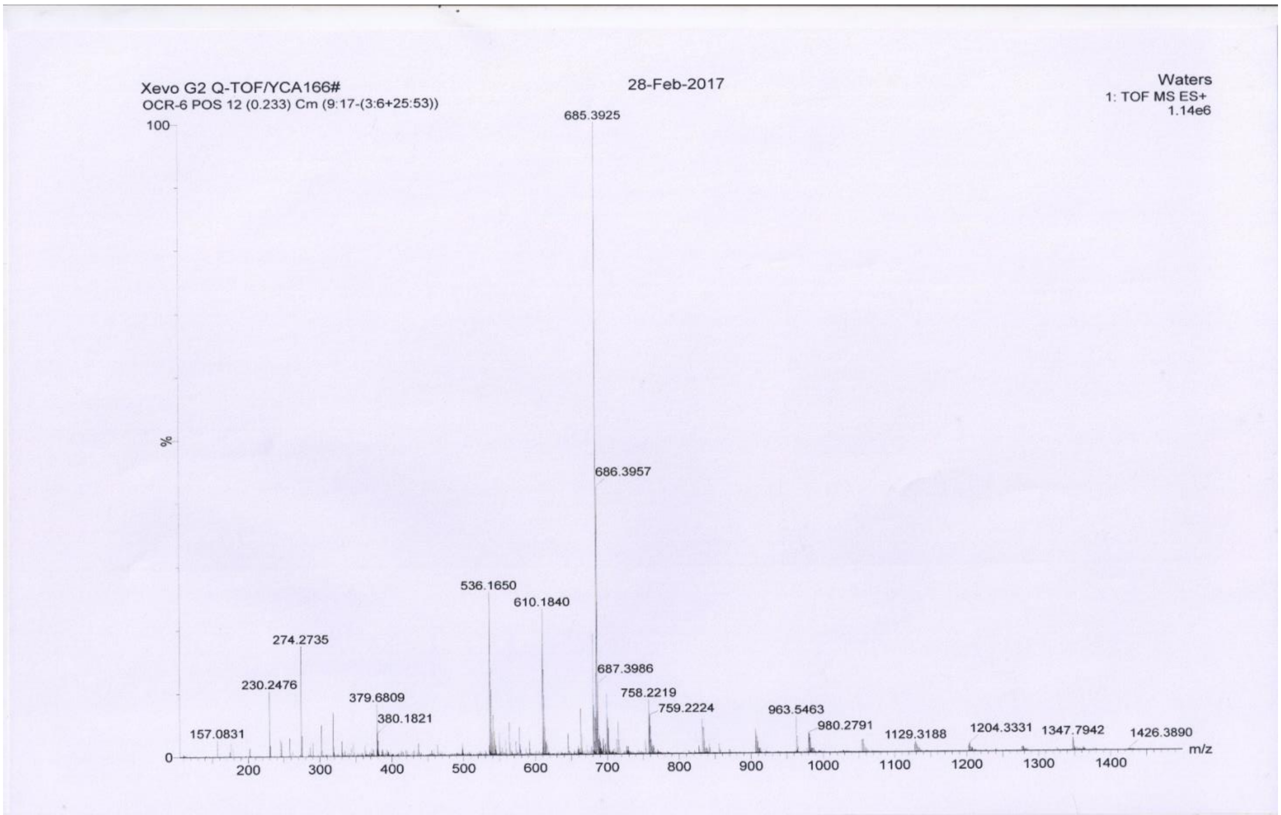

Figure S44. HRESIMS spectrum of myrioside B 6'-methyl ester (**7**)

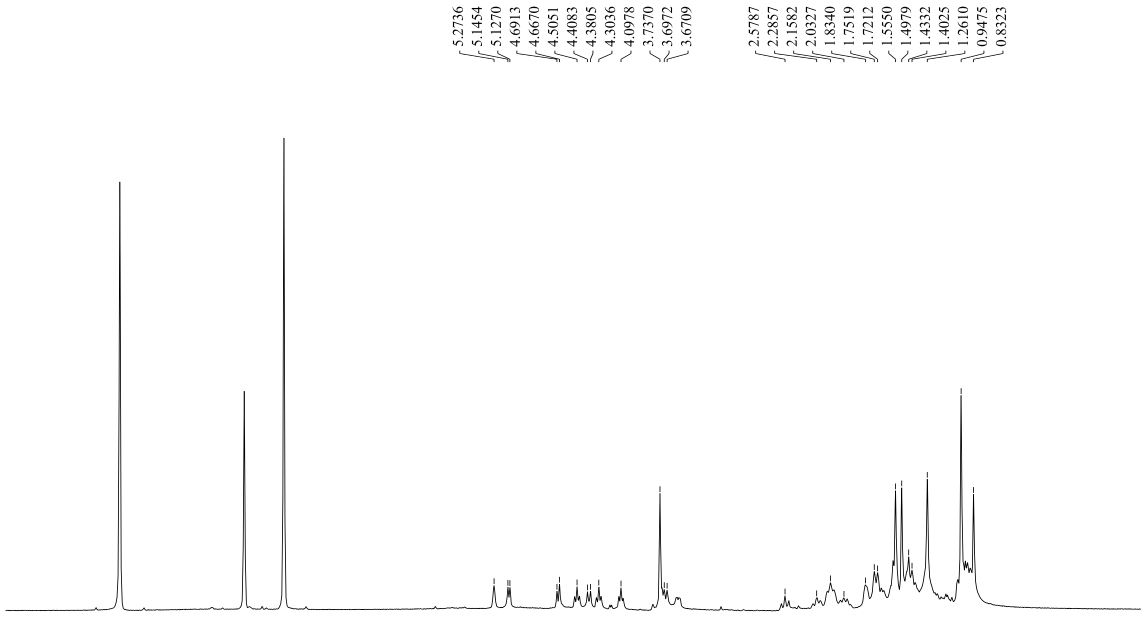

Figure S45. ^{1}H NMR spectrum of myrioside B 6'- methyl ester (**7**) in Pyr-d_5

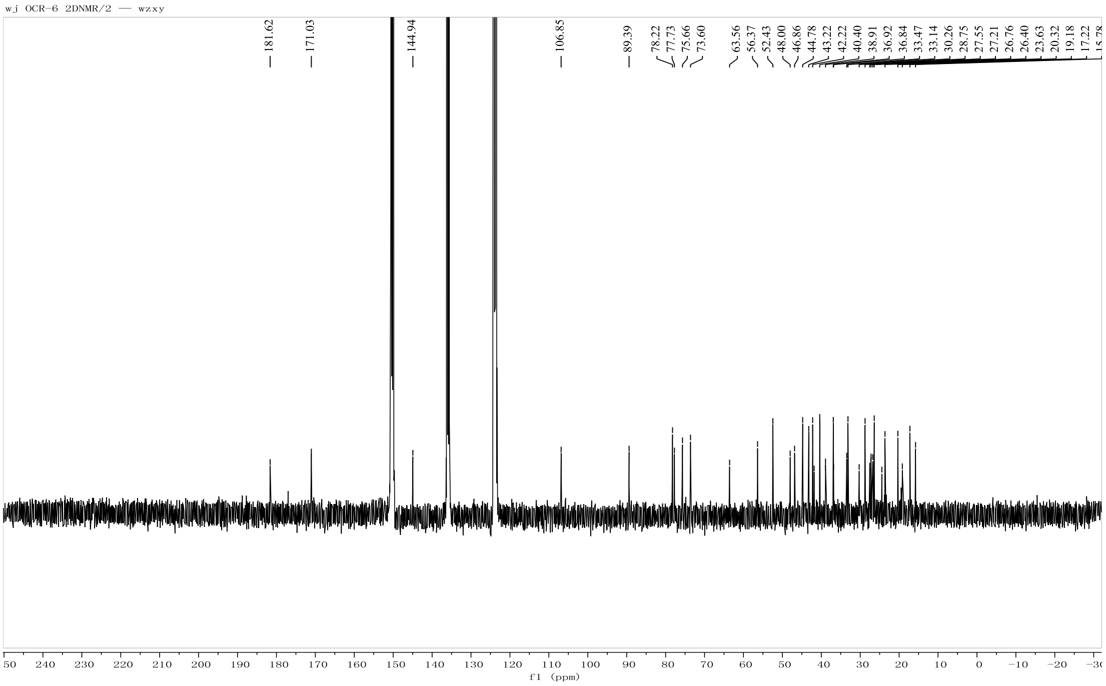

Figure S46. ^{13}C NMR spectrum of myrioside B 6'-methyl ester (**7**) in Pyr-d_5

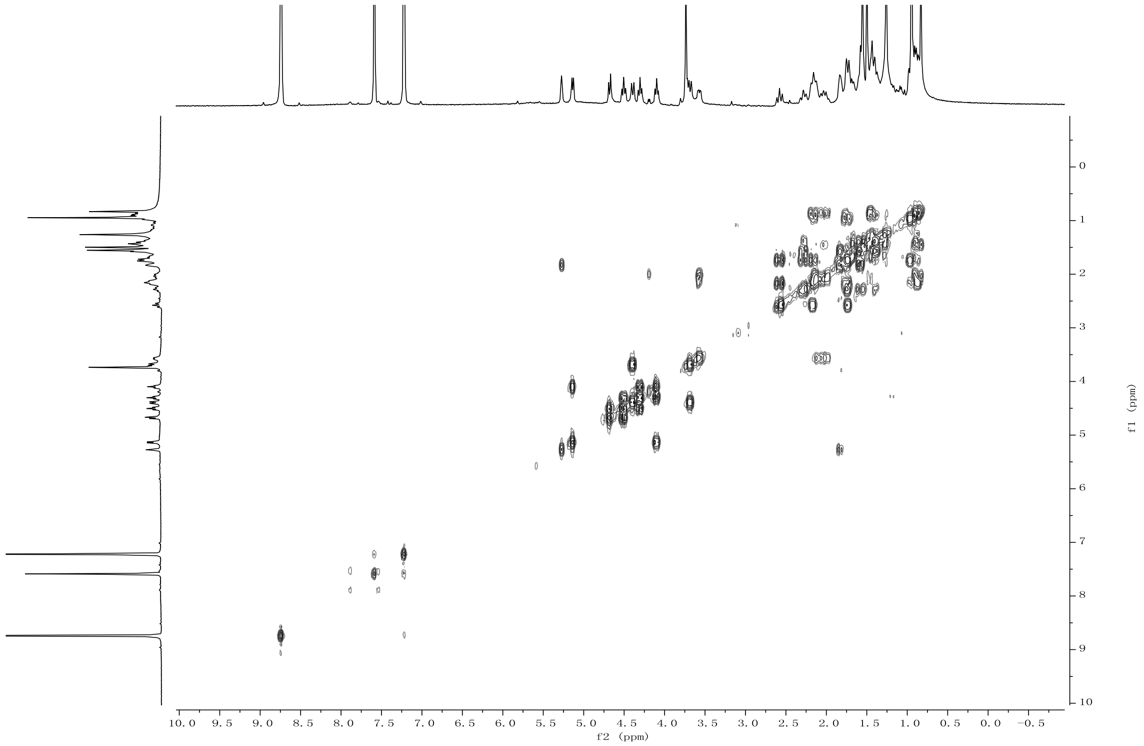

Figure S47. ${}^{1}H$-${}^{1}H$ COSY spectrum of myrioside B 6'-methyl ester (**7**) in Pyr-d_5

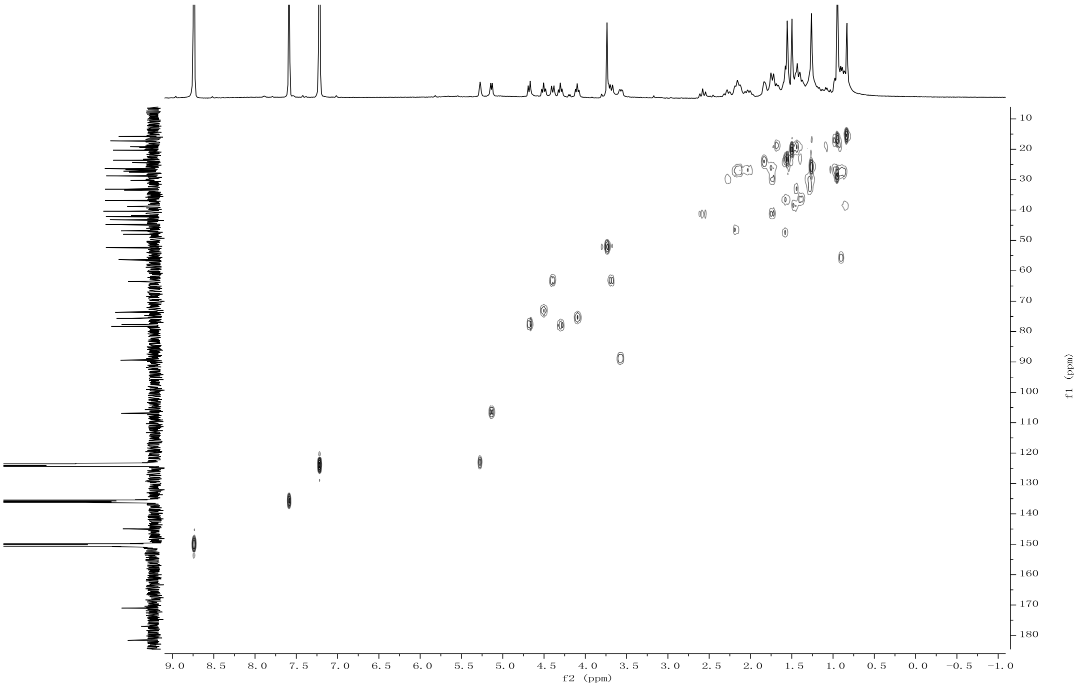

Figure S48. HSQC spectrum of myrioside B 6'- methyl ester (**7**) in Pyr-d_5

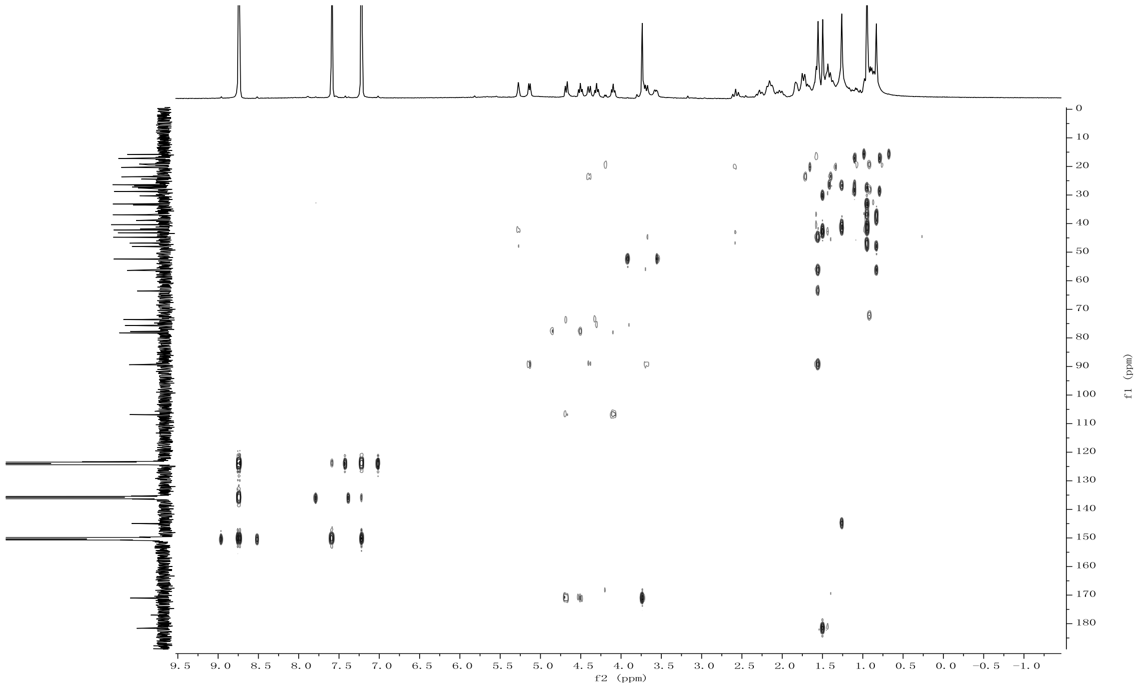

Figure S49. HMBC spectrum of myrioside B 6'-methyl ester (**7**) in Pyr-d_5
